# Adaptive morphological changes link to poor clinical outcomes by conferring echinocandin tolerance in *Candida tropicalis*

**DOI:** 10.1371/journal.ppat.1013220

**Published:** 2025-05-27

**Authors:** Yongqin Wu, Yun Zou, Yuanyuan Dai, Huaiwei Lu, Wei Zhang, Wenjiao Chang, Ying Wang, Zhengchao Nie, Yuanyuan Wang, Xiaohua Jiang

**Affiliations:** 1 Department of Laboratory Medicine, The First Affiliated Hospital of USTC, Division of Life Sciences and Medicine, University of Science and Technology of China, Hefei, Anhui, China; 2 Core Unit of National Clinical Research Center for Laboratory Medicine, Hefei, Anhui, China; 3 Unit of Pathogenic Fungal Infection & Host Immunity, Shanghai Institute of Immunity and Infection, Chinese Academy of Sciences, Shanghai, China; 4 University of Chinese Academy of Sciences, Beijing, China; 5 Hefei Center for Disease Control and Prevention, Hefei, Anhui, China; 6 The Center for Microbes, Development, and Health, Key Laboratory of Molecular Virology and Immunology, Unit of Pathogenic Fungal Infection & Host Immunity, Shanghai Institute of Immunity and Infection, Chinese Academy of Sciences, Shanghai, China; 7 Center for Reproduction and Genetics, Department of Obstetrics and Gynecology, The First Affiliated Hospital of USTC, Division of Life Sciences and Medicine, University of Science and Technology of China, Hefei, Anhui, China; University of Wisconsin Medical School, UNITED STATES OF AMERICA

## Abstract

Antibiotic tolerance, by which susceptible bacteria survive at high bactericide doses, is known to cause treatment failure in clinical practice. However, the impact of antifungal tolerance on clinical outcomes remains poorly understood. Here, we observed that candidemia cases caused by echinocandin-tolerant *Candida tropicalis* exhibited higher mortality rates during caspofungin treatment by conducting a comprehensive seven-year retrospective analysis. *C. tropicalis* develops tolerance to caspofungin by forming multicellular aggregates, a process linked to defects in cell division, both *in vitro* and *in vivo*. Our omics-based profiling results reveal that *C. tropicalis* develops tolerance through the intricate modulation of cell wall integrity and cell division pathways, particularly through the activation of chitin synthesis and the downregulation of cell division-related genes. The overexpression of cell division-related factor Ace2 can suppress the tolerance of *C. tropicalis* to caspofungin by delaying the formation of multicellular aggregates. Moreover, calcineurin inhibitors can suppress the tolerance of *C. tropicalis* by disrupting these adaptive molecular changes, thereby significantly enhancing the antifungal efficacy of caspofungin in a *Galleria mellonella* model. Collectively, our findings provide evidence that *C. tropicalis* acquires echinocandin tolerance through morphological alterations, and that inhibiting calcineurin may be a promising method to reduce this tolerance.

## Author summary

*Candida tropicalis* has emerged as a life-threatening bloodstream pathogen, particularly in the Asia-Pacific and Latin America regions, where escalating infection rates and azole resistance present growing clinical challenges. While echinocandins like caspofungin remain first-line therapies, treatment failures frequently occur despite susceptible *in vitro* test results. Our research uncovers why: *C. tropicalis* can survive treatment by forming multicellular aggregates through remarkable morphological adaptations, directly linking this tolerance mechanism to poor patient outcomes. We identified two central molecular drivers — cell wall remodeling and downregulation of cell division regulators like Ace2 — that enable this survival strategy. Most importantly, we demonstrated that both genetic restoration of Ace2 expression and pharmacological calcineurin inhibition can effectively reverse tolerance, providing the first definitive evidence that morphological plasticity mediates echinocandin treatment failure. These findings not only explain the troubling disconnect between laboratory susceptibility tests and clinical responses but also reveal actionable therapeutic targets, paving the way for life-saving combination therapies against resilient candidemia infections.

## Introduction

Candidemia, a type of bloodstream infection caused by *Candida* species, is one of the most common fungal sepsis occurrences with high rates of mortality, particularly among hospitalized patients with hematological disorders [1,2]. While *Candida albicans* remains the predominant pathogen, *Candida tropicalis* infections have exhibited a significant rise in morbidity and mortality rates, particularly in Asia-Pacific and Latin America regions [3–5]. Alarmingly, the resistance of *C. tropicalis* to azoles has also been on the rise, posing a significant challenge to the treatment of these infections in Asia-Pacific regions [2,4]. The fluconazole resistance rate of *C. tropicalis* isolates in mainland China has escalated dramatically from 5.7% to 31.8% [6]. Notably, azole-resistant isolates are predominantly clustered within a major resistant clone in terms of phylogenetic relationships, a pattern observed across both mainland China and Taiwan [7,8]. In addition, hotspot mutations and tandem duplications within the *ERG11* gene are potential drivers of the high-level resistance to azole antifungal agents in *C. tropicalis* [7]. Therefore, for *C. tropicalis* candidemia, azole drugs are apparently not suitable as the first-line treatment in some regions.

Echinocandins, including caspofungin and micafungin, which target the β-1,3-glucan synthase (Fks1), are currently the first-line antifungal agents for the treatment of candidemia [2]. *Candida* species generally exhibit high *in vitro* susceptibility to echinocandins, with over 98% of clinical isolates except *C. glabrata* showing sensitivity to both caspofungin and micafungin [4]. Mutations in the Fks1 hot spot region frequently confer resistance to echinocandins in *Candida* species [9]. Echinocandins, in the process of inhibiting 1,3-β-glucan synthase, also trigger cell wall stress responses, which are characterized by an increase in chitin content and enhanced exposure of 1,3-β-glucan in *Candida* species [10,11]. These processes are regulated by stress response pathways, including the calcineurin pathway, Hsp90, and the mitogen-activated protein kinase (MAPK) pathways [11,12]. Enhanced chitin synthesis in *Candida* species correlates with reduced susceptibility to caspofungin, but this diminished sensitivity can be countered by the use of calcineurin inhibitors, which enhance their response to the drug [13,14].

Antimicrobial tolerance, initially discovered in the context of antibiotic treatment for bacterial sepsis, involves complex mechanisms [15]. Recently, the concept of antifungal tolerance has been clarified: it is the ability of a drug-susceptible fungal strain to grow in the presence of an antifungal drug at concentrations that exceed the minimum inhibitory concentration (MIC) [13,16–18]. Moreover, this concept of tolerance must be refined for specific classes of antifungal drugs, distinguishing between fungistatic agents like azoles and fungicidal drugs such as echinocandins and amphotericin B. *Candida* species typically show high *in vitro* susceptibility to echinocandins, yet a recent European multicenter cohort study reported a candidemia-related mortality rate of 40.4%, with *C. tropicalis* infections being the most lethal, causing a 63.6% death rate [5]. This divergence between the promising *in vitro* susceptibilities and the poor clinical outcomes in treating candidemia indicates that there are many factors mediating the treatment of candidemia. Esteemed researchers propose that overlooked antifungal drug tolerance could be a contributing factor to the persistent candidemia and poor treatment outcomes [13,16]. It has been confirmed that the tolerance of *C. albicans* to fluconazole is an independent predictor of persistent candidemia [19,20]. However, the clinical correlation between antifungal drug tolerance and persistent candidemia remains poorly understood, primarily due to a lack of reports on the tolerance of *Candida* to echinocandins.

*Candida* species often adapts to the host environment by altering its morphology, which includes various forms such as yeast and hyphal states [21]. Multicellular aggregation is one of the adaptive strategies that *Candida* species employs in response to stress. In *Candida* auris, two types of multicellular aggregations have been observed: one that is dependent on ALS-family adhesins and another that results from defects in cell division, where daughter cells fail to detach from the mother cell post-budding [22]. In the presence of high concentrations of echinocandins, some clinical isolates of *Candida* species exhibit discontinuous growth, also known as paradoxical growth, during which some *Candida* species can form multicellular aggregate morphology [23]. In *C. auris*, these aggregates are induced by echinocandins due to defects in cell separation, whereas in *C. albicans*, the aggregation is dependent on adhesins [22,24]. In *C. auris* and *C. glabrata*, strains exhibiting multicellular aggregates have enhanced virulence and can withstand the attack of immune factors [25,26]. When exposed to echinocandin stress, certain *Candida* species strains exhibit a multicellular aggregation morphology while undergoing cell wall remodeling [27,28]. *C. albicans*, under cell wall stress, activates an alternative pathway for septum formation and cytokinesis, resulting in the development of a chain-like cellular morphology [29]. Genetic changes related to cell wall integrity, cytokinesis, and the regulation of Ace2 morphogenesis (RAM) pathway in *C. auris* are linked to the formation of multicellular aggregation [25]. Consequently, *Candida* exhibits adaptive morphological alterations in the face of survival challenges.

In clinical practice, we have observed cases where *C. tropicalis* isolates exhibited sensitivity to echinocandins *in vitro*, but treatment failures occurred with caspofungin therapy for *C. tropicalis* candidemia. Here we revealed that candidemia cases with *C. tropicalis* tolerance to echinocandins experience a heightened mortality when treated with caspofungin by a retrospective analysis spanning seven years. Using multi-omics profiling and genetic manipulation, we showed that *C. tropicalis* develops tolerance to caspofungin by forming multicellular aggregates. Moreover, we found that calcineurin inhibitors significantly reversed the tolerance of *C. tropicalis* to caspofungin in both *in vitro* and *in vivo* settings.

## Results

### Echinocandin-tolerant *C. tropicalis* contributes to caspofungin treatment failure

Among the 50 patients with *C. tropicalis* bloodstream infection, 36% (18/50) received prophylactic treatment with fluconazole or voriconazole (S1 Table). Furthermore, 60% (30/50) of these patients were neutropenic. The overall mortality rate was 40% (19/47) at 30 days from the onset of candidemia. Antifungal susceptibility testing on these isolates revealed that 46% (23/50) were resistant to both fluconazole and voriconazole, whereas all isolates demonstrated sensitivity to caspofungin and micafungin (S1 Table). Additionally, all isolates exhibited wild-type phenotype to amphotericin B, with geometric mean MIC value of 0.078 μg/mL for isavuconazole. Among the fluconazole-resistant isolates, 91% (21/23) harbored the Y132F and S154F double substitutions in Erg11, whereas one isolate did not exhibit any amino acid substitutions (S1 Table). Notably, caspofungin treatment failure (defined as persistent candidemia >10 days with positive blood cultures despite standard dosing: 70 mg loading dose followed by 50 mg/day maintenance) occurred in two patients infected with azole-resistant isolates SLT9 and SLT38 (fluconazole MIC = 128 μg/mL, S1 Table). Despite *in vitro* susceptibility to caspofungin (MIC ≤ 0.03 μg/mL) for these isolates, clinical resolution of the infections required a switch to amphotericin B therapy (S1 Table).

**Fig 1:**
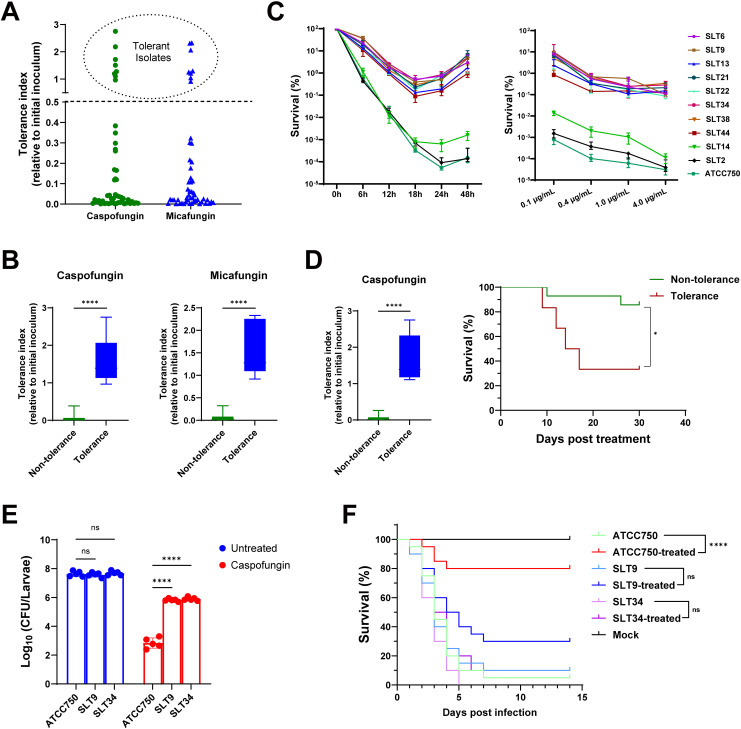
The tolerance of *Candida tropicalis* isolates to echinocandins contributes to the therapeutic failure of caspofungin *in vivo.* (A) Clinical isolates of *C. tropicalis* were cultured overnight, centrifuged, washed in PBS, and adjusted to a concentration of 2 × 10^5^ CFU/mL in YPD medium with 1μg/mL caspofungin or 1μg/mL micafungin. The cultures were then incubated at 37 °C for 24 hours. The tolerance index of each isolate was determined by comparing the CFU count to the initial inoculum. Isolates with a tolerance index exceeding 0.5 were classified as tolerant. (B) Statistical comparison of tolerance indices between the screened tolerant and non-tolerant strain groups under caspofungin (left panel) and micafungin (right panel) treatment was performed using the Mann-Whitney U test. The data are presented as the mean ± standard deviation (SD). (C) Both tolerant isolates and non-tolerant isolates, along with the reference strain ATCC750 were grown overnight in YPD medium, washed with PBS, and then inoculated at a density of 2 × 10^5^ CFU/mL into 2 mL YPD broth with or without caspofungin at 37 °C. Survival curves were constructed by normalizing the CFU counts of treated samples to those of untreated controls. Left panel represents the time course, while the right panel illustrates the dose-reponse relationship. (D) This survival analysis included 20 enrolled subjects with well-documented clinical data, who had experienced candidemia caused by a single pathogen and had received primary treatment with caspofungin for a minimum of one week without any evidence of concurrent infections from other pathogens. Subjects were categorized into "tolerance" and "non-tolerance" groups based on the echinocandin tolerance indices of the isolated strains as determined by the aforementioned results. Statistical comparison of tolerance indices between tolerance and non-tolerance groups following caspofungin treatment was performed using the Mann-Whitney U test (left panel). Survival data were analyzed using the Kaplan-Meier method, and statistical significance was assessed with the log-rank (Mantel-Cox) test (right panel). (E) Comparison of fungal burdens in *Galleria mellonella* larvae infected with tolerant strains and ATCC750, with or without caspofungin treatment. (n = 5 per group). Statistical analysis was performed using a two-way ANOVA. (F) Kaplan-Meier survival analysis of *G. mellonella* larvae (n = 20 per group) following infection with the tolerant strains or reference strain ATCC750 (inoculum: 1 × 10^6^ CFU/larva). Caspofungin-treated groups received 0.5 μg/larva at 1 h post-infection. Statistical analysis was performed using a log rank (Mantel-Cox) test. *, *P* < 0.05; ****, *P* < 0.0001; ns, not significant.

To evaluate echinocandin tolerance in clinical isolates, we employed a tolerance index (TI) to screen for echinocandin-tolerant strains, calculated as the survival fraction after 24-hour exposure to caspofungin or micafungin. Using a TI threshold >0.5, we identified eight tolerant isolates (Fig 1A and S1 Table). Notably, tolerant strains consistently displayed significantly higher TIs than non-tolerant strains for both caspofungin and micafungin (Fig 1B). Time-kill and dose-response analyses demonstrated persistent survival advantages in all tolerant isolates (SLT6, SLT9, SLT13, SLT21, SLT22, SLT34, SLT38, SLT44) relative to non-tolerant controls (SLT14, SLT2) and ATCC750 (Fig 1C). Tolerant isolates maintained stable survival rates across increasing caspofungin concentrations, in contrast to the dose-dependent viability decline in controls. This enhanced tolerance was further validated by spot growth assays comparing tolerant strains with ATCC750 (S1 Fig).

A retrospective cohort study was conducted to evaluate the clinical implications of *in vitro* caspofungin tolerance in 20 candidemia patients infected with *C. tropicalis*. Patients were stratified into tolerance and non-tolerance cohorts based on isolates' tolerance index. Strains from the tolerance cohort demonstrated significantly elevated TIs compared to non-tolerant counterparts (Fig 1D, left panel). Kaplan-Meier analysis revealed divergent 30-day survival outcomes (*P* = 0.011), with tolerance cohort patients demonstrating substantially lower survival rates (33%) compared to the non-tolerance cohort (85%) (Fig 1D, right panel). Notably, all surviving tolerance-group patients developed persistent *C. tropicalis* candidemia despite therapy, while none in the non-tolerance group showed recurrent infection (S1 Table).

To further validate that the *in vitro* observed strain tolerance constitutes a critical determinant of poor clinical therapeutic outcomes, we conducted systematic evaluations of both tolerant and non-tolerant strains using the *Galleria mellonella* larval infection model. Initial assessments demonstrated that larvae infected with tolerant strains SLT9 and SLT34 exhibited over 1,000-fold higher fungal burdens compared to those infected with the reference strain ATCC750 at 24h post-caspofungin treatment (Fig 1E). Survival analysis showed marked efficacy disparities: non-tolerant strains (ATCC750, SLT2, SLT14) exhibited 75–90% mortality reduction with caspofungin therapy, while six tolerant strains (SLT9, SLT34, SLT6, SLT21, SLT22, SLT44) showed no therapeutic response (Figs 1F and S2). Although caspofungin showed partial activity against tolerant strains SLT13 and SLT38 (15–20% mortality reduction), the effect remained substantially weaker than that observed against non-tolerant isolates (S2 Fig). These *in vivo* results conclusively demonstrate that the *in vitro* defined tolerance phenotype directly correlates with diminished therapeutic efficacy. We chose SLT34 and SLT9 for further mechanistic studies based on their contrasting phenotypes (highest vs. intermediate TIs, respectively) and clinical origins (breakthrough infection vs. persistent candidemia, respectively).

### Adaptive morphological changes in caspofungin-tolerant strains during caspofungin exposure *in vitro* and *in vivo*

**Fig 2:**
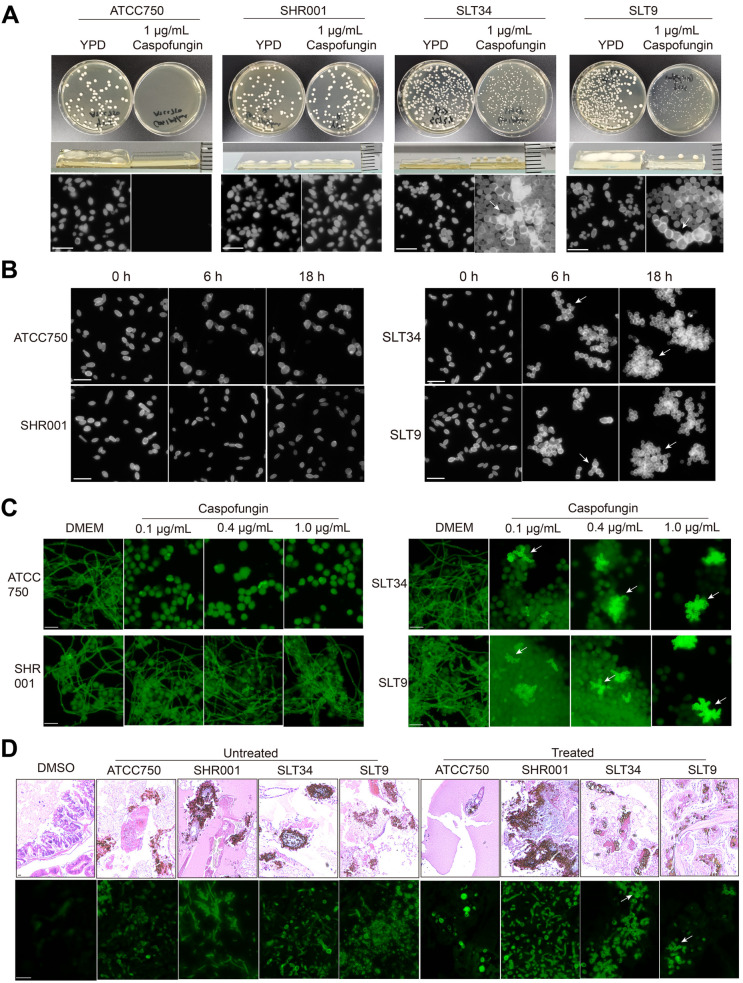
Multicellular aggregation in tolerant strains following caspofungin exposure, as observed in *vitro* and in *vivo.* (A) Cultures at the logarithmic growth phase of all strains were pelleted by centrifugation, rinsed with PBS, and then diluted before being spread onto YPD agar plates with and without 1ug/mL caspofungin. After a 48-hour incubation at 37 °C, images were captured. Subsequently, individual colonies were suspended in PBS, and stained with Calcofluor White (CFW) for microscopic analysis. ATCC750 is the reference strain, SHR001 is characterized as caspofungin-resistant, and SLT34 and SLT9 are classified as tolerant strains. The scale bar represents 20 µm. (B) Each strain was cultured in YPD liquid medium containing caspofungin, with an initial optical density (OD) of 2, and incubated at 37 °C with shaking. At 0, 6, and 18 hours' post-drug exposure, aliquots of the culture were taken, fixed with 4% paraformaldehyde, then stained with CFW, and observed under a fluorescence microscope. (C) The cell line RAW 264.7 was co-cultured with the strains at a multiplicity of infection (MOI) of 10, and various concentrations of caspofungin were added to the co-culture system. After an 18-hour incubation period at 37 °C, the samples were stained with CFW and then examined under a fluorescence microscope. (D) After a 48-hour infection of *G. mellonella* larvae with the strains, histopathological examination was performed. Tissue sections were stained using haematoxylineosin (HE) to assess general morphology and CFW to highlight specific cellular structures. White arrows indicate multicellular aggregate morphology. The scale bar represents 20 µm.

To further understand of the mechanisms by which echinocandin-tolerant isolates rapidly adapt and survive upon drug exposure, we initiated our investigation by employing population analysis profiling to screen for heteroresistance to caspofungin. No heteroresistance was detected, but we discovered atypical growth characteristics of the tolerant isolates on YPD plates containing caspofungin. Caspofungin-resistant strains produced creamy white, moist colonies on both drug-containing and drug-free plates. In contrast, all tolerant strains exhibited a delayed colony formation on the drug-containing plates, which occurred a day later, and the colonies were small, raised, and dry, composed of spherical cells and aggregated cells with abnormal divisions and enlarged size (Figs 2A and S3). Following caspofungin exposure in YPD medium, tolerant strains SLT9 and SLT34 developed multicellular aggregates due to incomplete cell division, retaining physical connections between mother-daughter cells (Fig 2B). These structures matured into three-dimensional grape-like clusters by 18 hours. Subsequent comprehensive analysis confirmed this aggregation phenotype in all remaining tolerant strains, demonstrating conserved stress adaptation (S4 Fig). In contrast, non-tolerant strains (ATCC750, SLT2 and SLT14) exhibited swollen, irregular, or even fragmented morphologies (Figs 2B and S4).

To rule out the possibility that cell adhesion was causing the aggregation, we treated the aggregated cells with trypsin and proteinase K, which resulted in no change; however, ultrasonic treatment successfully dispersed the cells (S5 Fig). Following exposure to caspofungin, we performed PI (propidium iodide) staining on the yeast cells of the strains and observed that approximately 80% of the ATCC 750 strain cells exhibited signs of death after 6 hours of caspofungin exposure (S6 Fig). In contrast, the tolerant strain showed less than 40% cell death even after 18 hours of exposure to caspofungin. Moreover, the dead cells were predominantly found within the aggregated cellular clusters, while the dispersed cells remained largely viable.

We further co-cultured the tolerant strains with macrophages at an MOI of 10 and observed that even at low drug concentrations, the tolerant strains exhibited aggregation, which became more pronounced under higher drug concentrations (Fig 2C). After extending the culture period, we did not observe hyphal growth of the tolerant strains. However, when co-cultured with macrophages at an MOI of 1, hyphal growth was evident in the tolerant strains after 48 hours (S7 Fig). To explore whether this aggregative morphology exists *in vivo*, we constructed a *Galleria mellonella* larval infection model. We found that in untreated larvae, all strains caused extensive infection foci, with hyphal invasion leading to widespread tissue damage (Fig 2D). In larvae treated with caspofungin, the tissue pathology of the caspofungin-resistant strain SHR001 group showed no significant difference compared to the untreated group. In ATCC750 group, the infection foci were smaller, and the yeast cells were enlarged and spherical; in tolerant strains SLT9 and SLT34 groups, the infection foci were dispersed, and the yeast cells aggregated, and exhibited abnormal divisions (Fig 2D). This suggests that during the course of caspofungin therapy, tolerant strains could exhibit a multicellular aggregate morphology *in vivo*.

**Fig 3:**
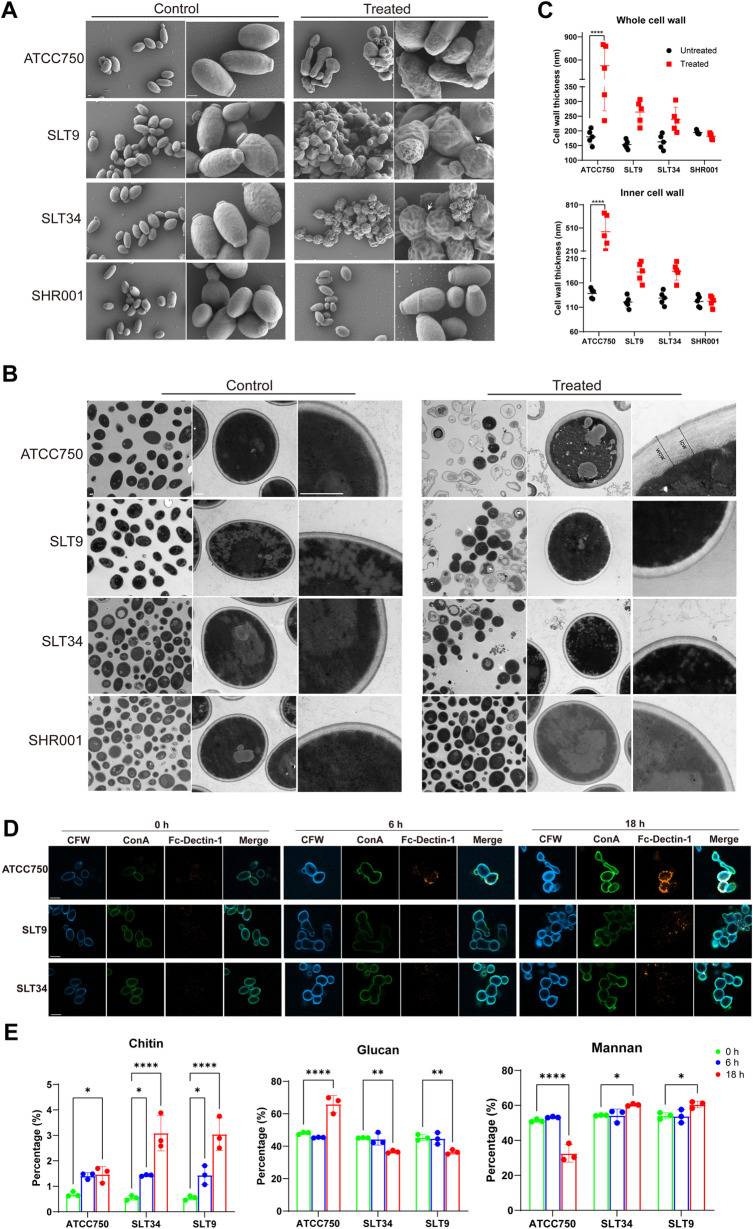
Changes in the cell wall of tolerant and non-tolerant strains under caspofungin treatment. Morphological examination of tolerant and non-tolerant strains under scanning electron microscopy (SEM) and transmission electron microscopy (TEM) (A-C). SEM images (A) and TEM images (B) are shown with scale bar as 1 µm. White arrows indicate intercellular septa. WCW (whole cell wall) and ICW (inner cell wall) indicate the measured regions of the cell wall structure. (C) Quantification of whole cell wall and inner cell wall thickness from TEM images. Cell wall measurements were performed using ImageJ with scale bar calibration. For each experimental group, five cells were randomly selected, and ten measurements were made from different sections of each cell. (D) Representative fluorescence images of the three main cell wall polysaccharide components in tolerant and non-tolerant strains under caspofungin stress at different time points. *C. tropicalis* cells were stained using ConA-FITC to delineate mannan, Fc-Dectin-1 to delineate β-glucan, and calcofluor white (CFW) to visualize chitin. The scale bar corresponds to 5 μm. (E) The monosaccharide composition of the cell wall in tolerant and non-tolerant strains under caspofungin stress, as determined by HPAEC-PAD. The values represent the percentage of each monosaccharide molecule, and these percentages are inferred to represent the polysaccharide components. The data are presented as the mean ± standard deviation (SD). Statistical analysis was performed using a two-way ANOVA. *, *P* < 0.05; **, *P* < 0.01; ****, *P* < 0.0001.

Given that the target of caspofungin is the glucan synthase enzyme in the fungal cell wall, we proceeded to further investigate the alterations in the cell wall of the tolerant strains under drug exposure. Scanning electron microscopy (SEM) revealed that under normal conditions, all strains exhibited an oval spherical cell morphology with smooth surfaces. Upon exposure to caspofungin, the ATCC750 cells were club-shaped, while the tolerant strains appeared as multicellular aggregates with wrinkled surfaces (Fig 3A). Transmission electron microscopy (TEM) more clearly confirmed that the multicellular morphology of the tolerant strains was caused by defects in cell division or budding processes (Fig 3B). Additionally, under caspofungin exposure, the ATCC750 cells contained an abundance of lipid droplets that had undergone fusion, with regions of the cytoplasm exhibiting dissolution. Furthermore, ultrastructural analysis revealed divergent cell wall remodeling strategies under caspofungin pressure. In the ATCC750 strain, we observed a significant 2.9-fold expansion in total cell wall thickness (from 179.6 to 526 nm; *P* < 0.001), accompanied by a 3.3-fold thickening of the inner cell wall layers (from 138.5 to 461.8 nm; *P* < 0.001) (Fig 3C). In contrast, the tolerant strains SLT9 and SLT34 exhibited only sporadic cell wall thickening in subpopulations of cells, with no statistically significant changes observed at the population level (Fig 3C). This phenotype divergence suggests tolerant strains may compensate through cell wall composition remodeling rather than bulk polysaccharide deposition.

The fungal cell wall is typically composed of mannans, glucans, and a minor fraction of chitin, with glucans and chitin constituting the primary components of the inner cell wall layer. We initially employed fluorescence staining to assess the variations in the composition of the cell walls of various strains. We observed that upon exposure to caspofungin, the ATCC750 strain exhibited a significant enhancement in the fluorescence signals of chitin and β-glucan over time, with a notable increase in the exposure of β-glucan (Fig 3D). In contrast, the tolerant strain showed a significant increase in chitin content, yet there was no apparent exposure of β-glucan (Fig 3D). This differential response may contribute to the tolerant strain's ability to evade immune recognition. Then the differences in polysaccharide constituents were detected using high performance anion exchange chromatography with pulsed amperometric detection (HPAEC-PAD). After a 6-hour exposure to caspofungin, an increase in chitin content within the cell walls was observed in all strains (Fig 3E). In contrast, following an 18-hour exposure, there was a significant rise (from 48.09% to 65.99%) in the relative proportion of glucan in the cell walls of the ATCC750 strain. Conversely, a significant reduction in glucan proportion was observed in the tolerant strains, with decreases from 45.15% to 36.59% for strain SLT34 and from 45.36% to 36.45% for strain SLT9 (Fig 3E). Notably, the HPAEC chromatograms of all strains were identical, with no extra peaks observed adjacent to the peaks corresponding to the three monosaccharides (S8 Fig), suggesting the absence of other monosaccharide derivatives.

### Omics-based profiling unveils adaptive molecular regulation of cell wall and cell division in caspofungin-tolerant isolates

It was reported that mutations in non-hotspot regions of the *FKS1* gene can lead to *Candida parapsilosis'* tolerance to caspofungin without conferring resistance [30]. Consequently, we sequenced the *FKS1* gene of all tolerant isolates, but no missense mutations were identified (S1 Table). To explore whether the tolerance to caspofungin observed in the isolates identified in this study is associated with the paradoxical growth of strains under echinocandins, we examined the paradoxical growth of all isolates in the presence of caspofungin. We found that 37.5% of tolerant strains exhibited paradoxical growth, and 30% of the non-tolerant isolates showed paradoxical growth under caspofungin (S1 Table).

To further investigate the genetic basis of tolerance, we performed whole-genome sequencing on representative strains, including non-tolerant strains ATCC750, SLT14, and the tolerant isolates SLT9, SLT13, SLT21, SLT22, and SLT34. Comparative genomic analysis revealed substantial heterogeneity among these strains, and no conserved mutations directly linked to tolerance were identified (S9 Fig). However, GO and KEGG analyses of homozygous mutations shared by these tolerant strains showed enrichment in the categories of "Carbohydrate metabolism" and "Cell cycle" (S9 Fig). While these findings do not establish direct causality, they suggest that tolerance may emerge through polygenic adaptation involving metabolic and cell wall remodeling systems.

**Fig 4:**
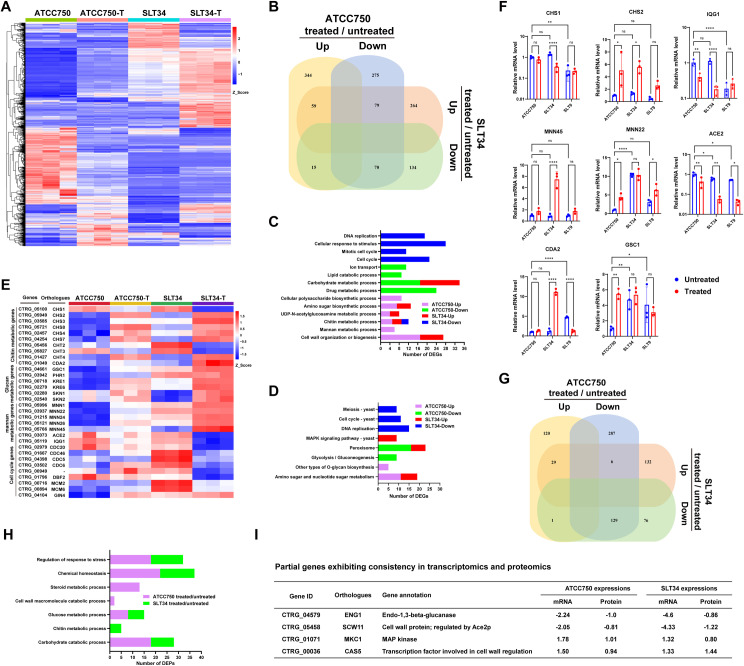
Role of cell wall integrity and cell division pathways in *C. tropicalis* tolerance to caspofungin. (A) Hierarchically clustered heatmap of RNA-seq expression profiles. ATCC750-T and SLT34-T represent strains treated with 1 μg/mL caspofungin for 1 h (n = 3 biological replicates per group). (B) A Venn diagram illustrating the differentially expressed genes following caspofungin treatment in the ATCC750 strain versus the SLT34 strain. (C) GO and (D) KEGG enrichment analyses are conducted on differentially expressed genes under caspofungin stress for both ATCC750 and SLT34 strains, where ATCC750-Up refers to the enrichment analysis outcomes for upregulated genes following caspofungin treatment in the ATCC750 strain, with other groups analyzed similarly. Selected terms and pathways are showcased. (E) Heatmap illustrating the expression levels of differentially expressed genes associated with cell wall integrity and cell cycle regulation across all groups. (F) mRNA levels of *CHS1*, *CHS2*, *MNN22*, *MNN45*, *GSC1*, *CDA2*, *ACE2*, and *IQG1* genes are quantified by RT-qPCR, with data normalized to the expression of the *ACT1* gene and presented relative to the untreated ATCC750 group. Data are expressed as mean ± SD and are representative of three biological replicates. Statistical analysis was performed using a two-way ANOVA with Tukey's test. *, *P* < 0.05; **, *P* < 0.01; ***, *P* < 0.001; ****, *P* < 0.0001. (G) Venn diagram comparing differentially expressed proteins between ATCC750 and SLT34 strains with or without caspofungin treatment, based on quantitative proteomic analysis. (H) GO enrichment analysis was performed separately on the differential expression proteins of the ATCC750 strain and the SLT34 strain after caspofungin treatment, with some GO terms displayed. (I) A table is presented showing some genes that have consistent expression patterns in both the transcriptome and proteome.

Subsequently, we selected the SLT34 strain and the control ATCC 750 strain for analysis using RNA-seq. Consistent with the substantial genomic variations, the mRNA expression profiles of the SLT34 strain and the ATCC70 strain also exhibit significant differences, with a total of 2,471 differentially expressed genes (DEGs) identified (Fig 4A and S2 Table). Under caspofungin stress, the ATCC750 strain shows a higher number of DEGs compared to the SLT34 strain (842 vs. 641), and the overlap of these DEGs is less than one-third (Fig 4B). Further GO enrichment analysis revealed that under caspofungin stress, genes upregulated in both the ATCC750 and SLT34 strains were significantly enriched in the GO terms "Cell wall organization or biogenesis" and "Chitin metabolic process" (Fig 4C). However, genes downregulated in the SLT34 strain were enriched in "Cell cycle", "Cellular response to stimulus", and "DNA replication", while those downregulated in the ATCC750 strain were enriched in "Carbohydrate metabolic process" and "Lipid catabolic process" (Fig 4C). Similarly, KEGG analysis indicated that under caspofungin stress, genes downregulated in the SLT34 strain were enriched in the "Cell cycle" and "DNA replication" pathways, whereas upregulated genes were enriched in the "MAPK signaling pathway" (Fig 4D). Analysis of the expression levels of genes related to cell wall polysaccharide metabolism and the cell cycle revealed that under normal conditions, the SLT34 strain exhibited significantly higher expression of cell wall-related genes, such as *CHT3*, *MNN22*, and *GSC1*, compared to the ATCC750 strain (Fig 4E). However, under caspofungin stress, the expression of these genes either decreased or showed no significant change. In contrast, the expression of cell cycle-related genes in the SLT34 strain, including *ACE2*, *IQG1*, and *DBF2*, significantly decreased under caspofungin stress (Fig 4E). Subsequently, we performed RT-qPCR analysis on a subset of genes associated with cell wall biosynthesis and the cell cycle, confirming that their expression patterns closely mirrored those observed in the RNA-seq data (Fig 4F). While the expression profiles of these genes in the tolerant strain SLT9 did not entirely match those of the SLT34 strain, the glucan synthesis gene *GSC1* was consistently found to be highly expressed under normal conditions across these tolerant strains, with minimal changes in expression under caspofungin stress (Fig 4F). Additionally, the cell division-related genes *ACE2* and *IQG1* in these tolerant strains showed a significant decrease in expression in response to caspofungin stress. To explore the relationship between genetic variations in the tolerant strain SLT34 and the distinct expression patterns of corresponding genes under caspofungin stress, we integrated transcriptomic and whole-genome sequencing data. This integration identified several genes associated with mitosis, such as *ENG1* and *SCW11*, which harbored homozygous indels (S3 Table). Notably, the expression levels of these genes exhibited a greater decrease in response to caspofungin stress compared to the ATCC 750 strain (S3 Table).

Subsequently, proteomic analysis was performed and revealed significant baseline protein expression differences between SLT14 and ATCC750, with 667 differentially expressed proteins (DEPs) identified (S4 Table). Under caspofungin stress, the ATCC750 strain exhibited 572 DEPs, whereas the SLT34 strain showed only 373 DEPs, and the overlap of upregulated DEPs between the two was less than a quarter (Fig 4G). Further GO enrichment analysis revealed that these DEPs are predominantly enriched in GO terms related to "Carbohydrate catabolic process", "Regulation of response to stress", and "Glucose metabolic process" (Fig 4H and S4 Table). Next, we conducted a concordance analysis of the proteomic and transcriptomic data and found that under caspofungin stress, only 54 DEGs in the ATCC750 strain and 55 DEGs in the SLT34 strain exhibited consistent expression patterns at both the transcriptional and protein levels, with very few overlapping DEGs between the strains (S5 Table). Further analysis of these genes revealed that cell division-related genes *ENG1* and *SCW11* showed a good consistency between transcription and protein expression (Fig 4I).

### Clinical isolates develop tolerance to caspofungin involving activation of chitin synthesis and downregulation of cell division-related genes

Based on our experimental results, it appears that the activation of chitin synthesis and the downregulation of cell division-related genes play a regulatory role in the tolerance of caspofungin by *Candida tropicalis* isolates. Nikkomycin Z, known for its inhibitory effect on chitin synthase enzymes, exhibits antifungal properties. We assessed the minimum inhibitory concentrations (MICs) of nikkomycin Z against ATCC750 and tolerant strains, revealing that the MICs consistently exceeded 16 μg/mL. However, the addition of nikkomycin Z significantly reduced the survival rates of tolerant strains under caspofungin, with a reduction in survival rates exceeding 10-fold (S10 Fig).

**Fig 5:**
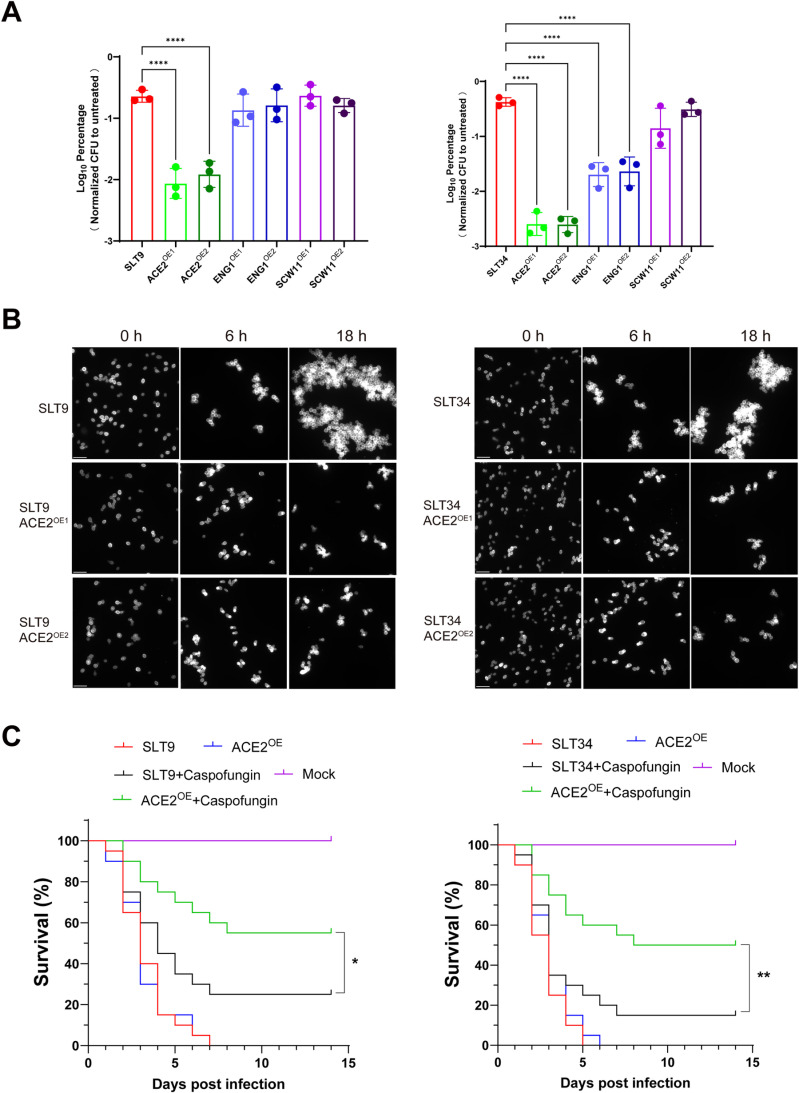
Overexpression of cell division-related genes partially restores caspofungin sensitivity in tolerant *C. tropicalis* strains. (A) Overexpression of cell division-related gene *ACE2* in tolerant strains significantly reduces fungal survival under caspofungin pressure. Following logarithmic phase growth, strains were adjusted to a density of 2 × 10^5^ CFU/mL in drug-supplemented YPD medium and incubated at 37 °C for 24 h. The yeast suspension was then diluted and 100 μL was spread onto YPD plates. After a 48-h incubation, CFUs were counted, and the survival rates were determined relative to the untreated control group. The data are presented as mean ± SD and are representative of three biological replicates. Statistical analysis was performed using a one-way ANOVA with Dunnett's test. ****, *P* < 0.0001. (B) *ACE2* overexpression delays the development of caspofungin-induced multicellular aggregates in tolerant strains. Each strain was cultured in YPD liquid medium containing caspofungin, with an initial optical density (OD) of 2, and incubated at 37 °C with shaking. Aliquots of the culture were taken, fixed with 4% paraformaldehyde, stained with CFW, and observed under a fluorescence microscope at 0, 6, and 18 hours' post-drug exposure. (C) In *Galleria mellonella* infection models, *ACE2* overexpression reverses therapeutic tolerance, as evidenced by improved larval survival following caspofungin treatment. Groups of larvae (n = 20 per group) were infected with 1 × 10^6^ CFUs of the respective strains and treated with or without caspofungin (0.5 μg/larva). Survival rates were assessed using Kaplan-Meier analysis, and statistical significance was determined using a log-rank (Mantel-Cox) test. *, *P* < 0.05; **, *P* < 0.01.

In order to further understand the mRNA expression characteristics of cell division-related genes identified by omics analysis in tolerant strains under caspofungin, we examined the mRNA expression of these genes at various time points after caspofungin treatment. We observed a significant reduction in the mRNA expression of the *ACE2* gene in the tolerant strains following caspofungin treatment, particularly in the strain SLT34, where a nearly 100-fold decrease was noted after 4 hours (S11A Fig). This dramatic response was not observed in non-tolerant strains, which showed minimal changes in *ACE2* gene mRNA levels post-treatment. Furthermore, in the tolerant strains SL9 and SLT34, the expression levels of both *ENG1* and *SCW11* genes plummeted by approximately 10-fold after caspofungin treatment (S11B and S11C Fig). This substantial decline in gene expression was maintained consistently over time. In contrast, the *IQG1* gene in the tolerant strain SLT9 did not exhibit any significant alterations in its expression following caspofungin treatment (S11D Fig). Subsequently, we overexpressed Ace2, Eng1, and Scw11 individually in caspofungin-tolerant clinical isolates (SLT9 and SLT34) and assessed their survival rates under caspofungin. We observed that the overexpression of Ace2 in the tolerant strains SLT34 and SLT9 not only delayed the formation of multicellular aggregates under caspofungin, but also significantly reduced the survival rates of the tolerant strains post-treatment (Fig 5A and 5B). Specifically, the survival rates of strains SLT34 and SLT9 decreased by 100-fold and 20-fold, respectively. Additionally, the overexpression of Eng1 significantly reduced the survival rate of the SLT34 tolerant strain post-treatment, with a reduction magnitude of approximately 10-fold. However, overexpression of Scw11 did not exhibit these effects. Furthermore, the overexpression of Ace2 in strains SLT9 and SLT34 significantly improved the recovery rate of the infected larvae in a *Galleria mellonella* model (SLT9: 55% vs 25%; SLT34: 50% vs 15%) (Fig 5C).

### Calcineurin inhibitors can reverse the tolerance of *C. tropicalis* to caspofungin both *in vitro* and *in vivo*

**Fig 6:**
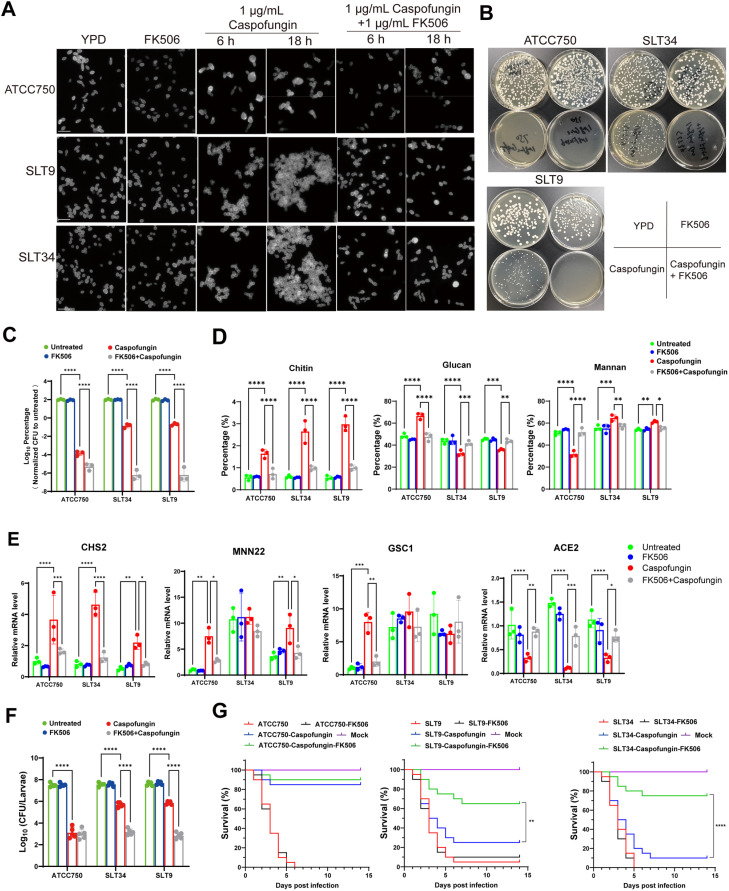
FK506 significantly potentiates caspofungin activity against tolerant *C. tropicalis* strains, reversing tolerance phenotypes in both *in vitro* and *in vivo* models. (A) FK506 co-treatment completely inhibited multicellular aggregate formation in tolerant strains at both 6 h and 18 h post-caspofungin exposure. The strains were subjected to treatments with 1 μg/mL caspofungin or 1 μg/mL FK506, followed by washing and staining with calcofluor white (CFW). The samples were then examined and captured under a fluorescence microscope, with the scale bar indicating 20 μm. (B) FK506 completely prevents colony formation of tolerant strains on YPD agar plates supplemented with 1μg/mL caspofungin. Strains were cultivated to the logarithmic phase, harvested, and diluted to suitable concentrations before being spread onto caspofungin-containing YPD plates and incubated at 37 °C for 48 h, after which photographs were taken. (C) FK506 significantly suppresses the survival of tolerant strains in YPD liquid medium supplemented with 1 μg/mL caspofungin. Following logarithmic phase growth, strains were adjusted to a density of 2 × 10^5^ CFU/mL in drug-supplemented YPD medium and incubated at 37 °C for 24 h. The yeast suspension was then diluted and 100 μL was spread onto YPD plates. After a 48-h incubation, CFUs were counted, and the survival rates were determined relative to the untreated control group. (D) FK506 effectively reverses the alterations in cell wall polysaccharides induced by caspofungin across different strains. Post-treatment with caspofungin or FK506, the cell walls were isolated, hydrolyzed to monosaccharides, and analyzed using high-performance anion-exchange chromatography with pulsed amperometric detection (HPAEC-PAD) to determine the monosaccharide composition, thereby inferring the polysaccharide composition of the cell wall in each strain. (E) FK506 counteracts the caspofungin-mediated adaptive gene expression changes in tolerant strains, as determined by RT-qPCR. The results were normalized to the expression levels of the gene *ACT1* and are presented as relative values compared to the untreated ATCC750 strain. (F) FK506 substantially decreases the fungal load in *G. mellonella* larvae infected with tolerant strains and treated with caspofungin. Larval tissues were homogenized, appropriately diluted, and 100 μL was spread onto YPD plates containing antibiotics. After a 24-h incubation, CFUs were counted and the number of CFUs per larva was calculated. (G) Kaplan-Meier survival curves were constructed for *G. mellonella* larvae post-infection with the designated strains and subsequent treatment with the specified drugs. Groups of *G. mellonella* larvae (n = 20 per group) were infected with 1 × 10^6^ CFUs of the respective strains and treated with caspofungin or FK506 (0.5 μg/larva). Survival rates were assessed using Kaplan-Meier analysis, and statistical significance was determined using a log-rank (Mantel-Cox) test. Statistical comparisons in (C-F) were performed using two-way ANOVA with Tukey's post-hoc test. *, *P* < 0.05; **, *P* < 0.01; ***, *P* < 0.001; ****, *P* < 0.0001.

In our quest to counteract the tolerance of *C. tropicalis* to caspofungin, we conducted a screening of various compounds to identify potential drugs that can reverse the tolerance. Fortunately, we have discovered that the calcineurin inhibitor FK506 is such potential drug. We observed that 1μg/mL of FK506 can completely inhibit the formation of multicellular aggregates by tolerant strains SLT9 and SLT34 exposed to caspofungin for 6 and 18 hours, respectively (Fig 6A). FK506 can suppressed the survival of all tolerant strains to caspofungin, exhibiting a concentration-dependent manner (S12 Fig). Upon the addition of 1 μg/mL FK506 to YPD plates containing caspofungin, it was found that the inclusion of FK506 could completely inhibit the formation of delayed growth small colonies by all tolerant strains under caspofungin stress (Figs 6B and S13A). Furthermore, the addition of FK506 markedly reduced the *in vitro* survival rate of all tolerant strains under caspofungin stress, by more than 10000-fold (Figs 6C and S13B). To determine whether the reversal of *C. tropicalis* tolerance to caspofungin is due to the inhibition of calcineurin or whether FK506 affects the tolerance through other pathways, we additionally conducted experiments to test the effect of another calcineurin inhibitor, cyclosporin A, in reversing the tolerance of *C. tropicalis* to caspofungin. Consistent with the effects observed following FK506 treatment, the treatment of cyclosporin A led to a reduction in the survival rate of tolerant strains under caspofungin stress, an effect that is concentration-dependent (S14A Fig). Specifically, a 10 μg/mL dose of cyclosporin A dramatically decreased the survival rate of the tolerant strains SLT9 and SLT34 by more than 10000 times under caspofungin. In addition, this concentration of cyclosporin A completely inhibited the formation of multicellular aggregate morphologies in strains SLT9 and SLT34 under caspofungin stress (S14B Fig).

Additionally, FK506 is capable of completely suppressing the increase in relative content of chitin and the decrease in relative content of glucan in the cell walls of tolerant strains exposed to caspofungin (Fig 6D). The RT-qPCR data demonstrate that caspofungin treatment significantly induces the expression of the chitin synthesis-related gene *CHS2* across all tested strains. Conversely, FK506 notably reverses the caspofungin induced elevation of *CHS2* mRNA levels in the ATCC750 strain and the tolerant strains SLT9 and SLT34 (Fig 6E). Furthermore, FK506 suppresses the elevation of mannan synthesis gene *MNN22* mRNA levels in the SLT9 strain, the upregulation of glucan synthesis gene *GSC1* mRNA levels in the ATCC750 strain, and the reduced expression of cell-division related gene *ACE2* mRNA levels in tolerant strains SLT9 and SLT34 under caspofungin (Fig 6E).

Next, we assessed the therapeutic potential of calcineurin inhibition in reversing caspofungin tolerance using a *Galleria mellonella* infection model. For non-tolerant strain ATCC750, caspofungin monotherapy achieved near-complete fungal clearance, with FK506 co-administration showing no additive benefit. For tolerant strains, co-treatment with FK506 and caspofungin significantly reduced the fungal burden in larvae compared to caspofungin alone. Specifically, the number of colony-forming units (CFU) recovered from larvae infected with tolerant strains SLT9 and SLT34 was reduced by more than 100-fold (Fig 6F). Additionally, the survival rate of larvae infected with these tolerant strains was markedly improved when treated with the combined administration of FK506 and caspofungin, compared to treatment with caspofungin alone. Specifically, for larvae infected with strain SLT9, survival rates increased by 40%, while those infected with strain SLT34 exhibited a survival rate increase of over 60% (Fig 6G). This suggests that the combination therapy provides a substantial therapeutic advantage over caspofungin alone in managing infections caused by echinocandin-tolerant strains.

## Discussion

*Candida* species' tolerance to antifungal agents poses a formidable challenge in the clinical management of candidemia, yet there is a paucity of clinical studies addressing this issue. The susceptibility data provided by clinical microbiology laboratories, which are based on the CLSI guidelines for broth microdilution methods, sometimes do not correlate with clinical outcomes when the isolates are reported as susceptible. This discrepancy is not solely attributed to pharmacokinetic factors but also to the intrinsic heterogeneity of *Candida* species and their potential for antifungal drug tolerance. In this work, we conducted a comprehensive analysis of the microbiological characteristics and clinical treatment data of clinical isolates of *C. tropicalis*. Our findings revealed that 16% of the *C. tropicalis* isolates exhibited tolerance to echinocandins, which was associated with a 50% increase in the 30-day mortality rate among the patients. Although our study included a limited number of patients, the data derived from clinical practice provide evidence for the relationship between antifungal drug tolerance and treatment outcomes.

In this study, both tolerant and non-tolerant strains exhibited an inducible increase in chitin content in their cell walls in response to caspofungin stress, which is consistent with previous reports [11,12]. This stress-induced elevation of chitin is a protective response that maintains the integrity of the cell wall structure. Therefore, treating tolerant strains with the chitin synthase inhibitor nikkomycin Z results in a significant reduction in their survival rate under caspofungin stress, yet the decrease remains modest. This indicates that the tolerance of these strains to caspofungin is mediated by a complex array of molecular regulatory mechanisms. It has been reported that caspofungin enhances the exposure of β-1,3 glucan in the cell wall of *C. albicans*, which can aid the immune system in clearing the fungus [11,31]. Our study similarly observed that caspofungin increases β-1,3 glucan exposure in non-tolerant strains of *C. tropicalis*. However, this increased exposure was not observed in tolerant strains, suggesting that the varied response to caspofungin among *C. tropicalis* strains could reveal new mechanisms behind persistent candidemia. Additionally, we observed that caspofungin can significantly reduce the glucan content in the cell walls of tolerant strains, but leads to a significant increase in glucan content in the cell walls of non-tolerant strains. Stevens *et al.* reported that under caspofungin treatment, a strain of *C. albicans* exhibiting paradoxical growth showed a decrease in β-glucan content in its cell wall of more than 70% compared to the untreated condition [32]. This suggests that the adaptive reduction of glucan content in response to caspofungin treatment may be a survival strategy for strains under caspofungin stress. Nevertheless, the survival-related adaptive alterations in the cell walls of tolerant strains are entirely suppressible by calcineurin inhibitors in this study. It's axiomatic that calcineurin, a regulator of stress responses, plays a role in a wide array of physiological processes, notably the modulation of cell wall integrity pathways [12,33].

Multicellular aggregate morphology is a distinctive form exhibited by yeasts under environmental stress, with defects in mother-daughter cell separation being one such type [34]. In this work, we observed for the first time that some *C. tropicalis* isolates, under caspofungin stress, forms multicellular aggregates with defects in mother-daughter cell separation, both *in vitro* and *in vivo*. Within these multicellular aggregates, some cells are dead, while scattered individual spores are often viable. Does this imply that cells at the core of the aggregate experience less drug pressure and could be the seeds of tolerant cells? Of course, this warrants further in-depth research. Cells exhibiting multicellular aggregate morphology cannot be cleared by macrophages [22]; similarly, in our study, we observed that the larger size of multicellular aggregates may prevent them from being engulfed by macrophages. In a neutropenic murine model of *C. auris* bloodstream infection, large aggregates were reportedly observed in the livers, kidneys, and hearts of the mice [35]. In this study, we similarly observed the formation of multicellular aggregates of *C. tropicalis* in tissues of *Galleria mellonella* larvae infected with *C. tropicalis* isolates and treated with caspofungin. Additionally, Bing *et al.* have reported that during systemic infection in mice, *C. auris* undergoes adaptive genetic mutations that lead to the formation of multicellular aggregates, which resist host antimicrobial peptides and facilitate its survival within the host [25]. Therefore, the multicellular aggregates can resist both drug stress and host immune attacks, which may be an important reason for the persistence of candidemia.

Based on the above discussion, we propose that the formation of multicellular aggregates may be a crucial factor for the survival of tolerant strains under caspofungin stress. Consequently, we conducted an in-depth analysis of the multi-omics data and discovered that the expression levels of cell division-related factors, including Ace2, Iqg1, Eng1, and Scw11, were decreased in tolerant strains when subjected to caspofungin stress. The transcription factor Ace2 is pivotal in initiating the expression of genes associated with the separation of mother-daughter cells during late cytokinesis, while Iqg1 regulates the assembly and contraction of the actomyosin ring [36,37]. Additionally, Eng1 and Scw11 are hydrolases that play a role in the efficient degradation of the septum [38]. Our RT-qPCR analysis revealed that with the extension of caspofungin treatment duration, the mRNA levels of Ace2, Eng1, and Scw11 progressively decreased. Notably, Ace2 mRNA expression showed a particularly pronounced reduction, even resulting in silencing. In *C. auris*, the functional loss of Ace2, Eng1, and Iqg1 each independently results in the formation of multicellular aggregates [25,39]. Consequently, we hypothesize that the downregulation of these genes following caspofungin treatment could be a potential cause for the formation of multicellular aggregates in tolerant strains. Subsequently, we overexpressed these genes in the tolerant strains and observed that the overexpression of the *ACE2* gene caused a delay in the formation of multicellular aggregate morphology under caspofungin, as compared to the wild-type strain. Furthermore, the survival rate of the mutant strain significantly decreased under caspofungin. These findings imply that the formation of multicellular aggregates may confer a survival advantage to tolerant strains under caspofungin, indicating that this morphological adaptation is a crucial determinant for their resilience to such pressures.

Our study identified calcineurin inhibitors (FK506 and cyclosporin A) as effective disruptors of caspofungin tolerance in *C. tropicalis*. In the *Galleria mellonella* model, FK506 combination therapy significantly enhanced survival rates compared to caspofungin monotherapy (60–75% vs 10–20%; *P* < 0.01), but the effect was less pronounced than *in vitro* observations. Three key factors likely contribute to the observed efficacy gap: (1) compensatory stress responses during disseminated infection, (2) suboptimal drug pharmacokinetics in the larval model, and (3) complex host-pathogen interactions absent in planktonic cultures. Notably, while calcineurin's evolutionary conservation presents challenges for targeted therapy [40], the consistent potentiation observed across experimental systems strongly supports its mechanistic role in tolerance. These findings provide a conceptual framework for future translational development, highlighting two critical directions: (1) optimization of drug delivery systems to overcome pharmacokinetic barriers, and (2) identification of downstream calcineurin effectors that could serve as more fungus-specific targets.

**Fig 7:**
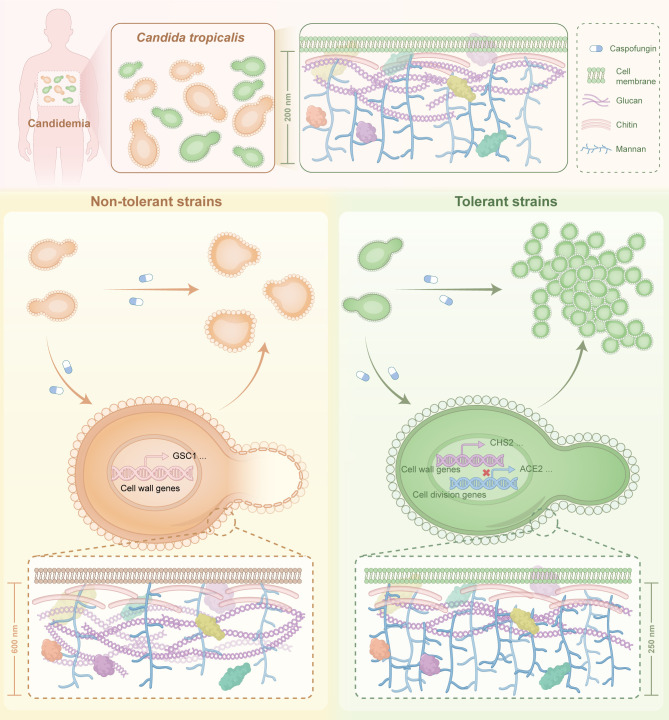
Proposed model of how morphological plasticity contributes to echinocandin tolerance acquisition in *C. tropicalis* clinical isolates. Upon exposure to caspofungin, tolerant strains rapidly form multicellular aggregates due to cellular division defects, accompanied by an increase in chitin content and a decrease in glucan content in the cell wall. In contrast, non-tolerant strains swell or even rupture, accompanied by a significant thickening of the cell wall, and a marked increase in both chitin and glucan content. Throughout this process, intricate alterations occur in the expression of genes (e.g., *CHS2* and *GSC1*) associated with the cell wall in both tolerant and non-tolerant strains, with a significant downregulation of genes (e.g., *ACE2*) related to cell division in the tolerant strains.

In conclusion, our study indicates that *C. tropicalis* develops tolerance to echinocandins through morphological changes, which can result in therapeutic failure. We have proposed a model to elucidate the characteristics of tolerant and non-tolerant strains, as shown in Fig 7. Nevertheless, the regulatory mechanisms underlying the rapid morphological adaptations in echinocandin-tolerant strains following caspofungin exposure remain poorly characterized. Our findings demonstrate that tolerant strains exhibit a significant survival advantage under antifungal pressure through multicellular aggregation and cell wall remodeling. Future studies will investigate host immune responses to these tolerant morphotypes and elucidate the molecular mechanisms governing their persistence during infection.

## Materials and methods

### Ethics statement

The study protocol was approved by the Medical Ethics Committee of the First Affiliated Hospital of University of Science and Technology of China (Permit Number: 2023-RE-346). Given the retrospective and observational nature of the study, the requirement for informed consent was waived.

### Patients

Clinical data pertaining to patients diagnosed with *C. tropicalis* bloodstream infections were meticulously collected by accessing the electronic medical records database of the First Affiliated Hospital of University of Science and Technology of China from January 2017 and May 2023. Patients were included in this survival analysis if they met the following criteria: (a) they had at least one positive blood culture for *C. tropicalis*, (b) there was no concomitant isolation of bacteria or fungi from blood culture within two weeks from the first date of candidemia, (c) there was no evidence of concurrent infection with other pathogens within 48 hours of the first date of candidemia, and (d) they received primary treatment with caspofungin, which was administered for at least 7 days. The primary endpoint of this study was the assessment of 30-day survival rates.

### Clinical isolate identification and growth

A collection of 50 clinical isolates of *C. tropicalis* was procured from 50 patients diagnosed with *C. tropicalis* bloodstream infections at the First Affiliated Hospital of the University of Science and Technology of China, spanning the period from January 2017 to May 2023. Blood samples for culture were collected from hospitalized patients, and yeast growth was identified using the BacT/Alert automated detection system (bioMérieux, France). The identity of each isolate as *C. tropicalis* was verified through Matrix-Assisted Laser Desorption/Ionization Time-of-Flight Mass Spectrometry (MALDI-TOF/MS; Vitek MS, bioMérieux) or sequencing of the internal transcribed spacer (ITS) region. All isolates were grown on Sabouraud or YPD plates at 30 °C.

### Antifungal susceptibility testing

The minimum inhibitory concentrations (MICs) of antifungal drugs were ascertained for 50 clinical isolates of *C. tropicalis* utilizing the broth microdilution technique, in strict accordance with the Clinical and Laboratory Standards Institute (CLSI) M27/4th edition guidelines. The antifungal agents assessed encompassed fluconazole, voriconazole, isavuconazole, micafungin, caspofungin, and amphotericin B, all procured from Selleck Chemicals (Houston, TX, USA). The MIC data for these drugs were interpreted in light of the CLSI documents M27M44S-Ed3 and M57S-Ed4. Concurrently, stringent quality control measures were implemented for each set of drugs, employing *Candida parapsilosis* ATCC 22019 and *Candida krusei* ATCC 6258 as reference strains. In the detection of paradoxical growth, the concentration range of caspofungin was from 0.125 to 64 μg/mL, and paradoxical growth was defined as growth resurgence in at least two dilution wells above the MIC after 48 hours of incubation.

### Echinocandins tolerance assessment

We initially screened for echinocandin tolerance by assessing survival following exposure to caspofungin and micafungin. In brief, all clinical isolates were cultured overnight in YPD medium, centrifuged, and washed with PBS. Fungal suspensions were standardized to 2 × 10^5^ CFU/mL in fresh YPD medium containing either 1 μg/mL caspofungin or micafungin. We selected this concentration because it represents the clinical resistance breakpoint for *C. tropicalis*. Cultures were then incubated at 37 °C with shaking at 220 rpm for 24 hours. Cells were subsequently harvested by centrifugation, washed three times with PBS, and plated on YPD agar for CFU counting. The tolerance index was calculated as the ratio of post-treatment CFU counts to the initial inoculum. Isolates exhibiting a tolerance index greater than 0.5 were categorized as tolerant. The selection of the cutoff for defining tolerant strains was based on the clear bimodal distribution observed across all clinical isolates, with minimal overlap between the tolerant and non-tolerant populations.

To validate the tolerance phenotypes identified in our initial screening, we performed standardized time-kill assays on all tolerant isolates along with the selected non-tolerant clinical isolates and the reference strain ATCC750. In brief, following overnight growth in YPD medium, cultures were washed with PBS and standardized to 2 × 10^5^ CFU/mL in fresh YPD broth with or without caspofungin supplementation. For time-dependent killing assessment, cultures were incubated at 37°C with agitation at 220 rpm, and aliquots were collected at 6, 12, 18, 24, and 48 hours' post-inoculation. The aliquots were serially diluted and plated on YPD agar for CFU counting. For dose-response assessment, cultures were treated for 24 hours under identical conditions. To ensure accurate quantification of aggregated cells, samples were sonicated for 50 s using an ultrasonic homogenizer (HD 2070.2, WIGGENS, Germany) prior to serial dilution and plating. Survival percentages were calculated relative to untreated controls at each point. All experiments included three biological replicates.

### *Galleria mellonella* model for *in vivo* therapeutic experimentation

The *Galleria mellonella* infection model was established following established protocols for studying *C. tropicalis* pathogenesis and antifungal drug efficacy [41]. *G. mellonella* larvae, exhibiting a length of 2.5 to 3.0 cm and a weight of 0.25 to 0.3 g, were procured from a commercial supplier (Keyun Biotechnology Company, China). Upon acquisition, the larvae were subjected to a stringent health assessment. Subsequently, the larvae were randomly assigned into multiple experimental groups, with 20 larvae in each group. Larvae were inoculated via the last left proleg with 10 μL of *C. tropicalis* suspension (1 × 10^6^ CFU/larva), a site chosen for its direct access to the insect's hemocoel, an open circulatory system where hemolymph bathes all internal organs. This injection method delivers fungal cells directly into the hemolymph, the functional equivalent of mammalian blood, enabling whole-body dissemination post-infection [42]. For the antifungal therapeutic protocol, the larvae were injected into the last right proleg with caspofungin (0.5 μg/larva) after 1 hour of infection. The uninfected larvae (Mock group) were used as a control group that received an equivalent volume of the drug. The larvae were incubated at 37 °C, with daily survival assessments conducted over a 14-day period. These experiments were meticulously repeated on at least two occasions.

Furthermore, the fungal burden within the larval tissue was assessed 24 hours' post-infection, with five larvae per group. Specifically, individual larvae were subjected to homogenization using a FastPrep-24 5G apparatus (MP Biomedicals, Shanghai, China) with a speed of 6.0 m/s for 40 sec. The homogenates were serially diluted and aliquots were spread onto YPD agar plates. Subsequently, the plates were incubated at 35°C for 24 hours. After incubation, the colonies were counted to determine the number of colony-forming units per larva (CFU/larva). The fungal burden in the larvae was then expressed as CFU/larva. For histopathological examination, three larvae of each group were fixed in a 4% formaldehyde solution for 30 days and the histological sections were stained using haematoxylineosin (HE) and Calcofluor white (CFW).

### Scanning electron microscopy (SEM) and Transmission electron microscopy (TEM) analysis

Yeast cells were collected and fixed in a solution of 2.5% glutaraldehyde in 0.1 M sodium cacodylate buffer (pH 7.4) at 4 °C for 2 hours to maintain cell integrity. Post-fixation, the samples underwent a dehydration process using an ethanol gradient (30% to 100%). Dehydrated samples were then subjected to critical point drying to eliminate the ethanol and any associated water content. Following this, the samples were sputter-coated with a thin layer of gold-palladium to enhance conductivity and image quality. The sputter-coated samples were examined using a scanning electron microscope (Hitachi, Japan) operated at an accelerating voltage of 10–20 kV. SEM images were obtained at various magnifications to assess the surface morphology of the yeast cells.

For TEM analysis, yeast cells were postfixed with 1% osmium tetroxide, dehydrated through an ethanol series, and infiltrated with an epoxy resin. Ultrathin sections were cut using an ultramicrotome (Leica, Germany) and collected onto copper grids. The ultrathin sections were stained with uranyl acetate and lead citrate to increase contrast and visualize cellular details. The stained sections were then examined under a transmission electron microscope at an accelerating voltage of 80–100 kV. TEM images were captured to elucidate the ultrastructure of the yeast cells. The measurement of cell wall thickness was conducted as described previously [43]. In brief, the cell wall thickness was systematically measured from TEM images using ImageJ with scale bar calibration. For each experimental group, five cells were randomly selected, and ten measurements were made from different sections of each cell.

### Cell wall composition analysis

The cell walls were extracted following a previously described protocol [43]. Briefly, strains were inoculated and grown overnight, then diluted 1:100 into fresh medium. Subsequently, the cultures were exposed to either PBS or 1 μg/mL caspofungin for 6 and 18 hours, respectively, at 37 °C with constant shaking. The yeast cells were harvested, washed, and resuspended in sterile water before being lysed using a FastPrep-24 5G cell disrupter apparatus (MP Biomedicals, Shanghai, China). The resulting pellet was washed with 1 M NaCl and then treated with a protein removal buffer by boiling for 10 minutes. After this, the cell wall was rinsed with water and subjected to freeze-drying using liquid nitrogen. Accurate weighing of 5 mg of the lyophilized cell wall powder was carried out. Following hydrolysis to monosaccharides, the samples were analyzed using high-performance anion-exchange chromatography with pulsed amperometric detection (HPAEC-PAD) on a Dionex ICS 5000 system (Thermo Scientific, American). The monosaccharide composition of the cell wall polysaccharides, including glucan, chitin, and mannan, was quantified and the respective polysaccharide content within the cell wall was determined.

### Fluorescence staining and confocal microscopy imaging of cell wall polysaccharides

The staining of cell wall polysaccharides was conducted according to a previously established protocol [43]. The strains were cultured overnight and then transferred to fresh medium for further incubation. Subsequently, the cultures were treated with either PBS or 1 μg/mL caspofungin for 6 and 18 hours, respectively. The cells were harvested and washed with PBS. And then the cells were fixed in 4% paraformaldehyde overnight at 4 °C. For mannan labeling, the fixed cells were washed with PBS and stained with Concanavalin A (ConA; Thermo Fisher Scientific, C11252) for 1 hour at 37 °C in the dark. β-glucan labeling involved a post-fixation wash with PBS, followed by staining with Fc-hDectin-1a (InvivoGen, fc-hdec1a) at 4 °C overnight. An Alexa Fluor 555-conjugated anti-human IgG antibody (Invitrogen, A-21433) was utilized as the secondary antibody, applied to the primary-stained yeast cells for 1 hour at 37 °C in the dark. Chitin was identified using calcofluor white (CFW; 30 μg/mL) with a 10-minute incubation at 37 °C in the dark. After staining, the cells were centrifuged and washed three times in PBS. The stained cells were then visualized with a confocal laser scanning microscope (Zeiss LSM800, Germany), and the images were captured and analyzed using ZEN Blue Lite software.

### *ERG11* and *FKS1* sequencing

Genomic DNA was extracted from the target strains, and the *ERG11* gene was amplified using the primers ERG11F (TTTGATTTATCACAGTTATAGACCC) and ERG11R (TGTATACTGTATTAAAGGCATAAATATG). Similarly, the *FKS1* gene was amplified with the primers FKS1F (TTTATTTCAGGACACACCCACAC) and FKS1R (AACAAAGAACCAGTGGAAATGATTT). The purified PCR products were then sent to Tsingke Biotechnology Co., Ltd. (Nanjing, China) for bidirectional Sanger sequencing. The resulting sequences were aligned with the *ERG11* reference sequence (GenBank: XM_002550939.1) and the *FKS1* reference sequence (GenBank: EU676168.2) for comparative analysis.

### Whole-genome sequencing (WGS)

The total genomic DNA of the strains was extracted and purified, followed by library preparation using Illumina's TruSeq DNA PCR-free prep kit according to the manufacturer's protocol. Library quality was evaluated with the Agilent High Sensitivity DNA Kit on an Agilent Bioanalyzer instrument. Libraries that met the quality criteria were then diluted in a gradient and mixed proportionally based on the desired sequencing depth. Following denaturation with NaOH to produce single-stranded DNA, the libraries were sequenced on the Illumina NovaSeq 6000 platform using paired-end sequencing.The raw sequencing data underwent quality control using fastp, an open-source tool designed to efficiently process and filter raw sequencing data to generate high-quality sequences. After quality filtering, the high-quality sequences were aligned to the reference genome (*Candida tropicalis* MYA-3404, GCF_000006335.3) using the bwamem algorithm with parameters set to default. SNP detection was conducted using the GATK software suite, while ANNOVAR was employed for annotating both SNP and InDel sites. Copy number variations (CNVs) across the genome were identified using the CNVnator software. The entire sequencing and analysis workflow was facilitated by Shanghai Personal Biotechnology Co., Ltd.

### RNA-seq and bioinformatics analysis

The strains ATCC750 and SLT34 were incubated at 37 °C for 1 h, with and without the addition of caspofungin. RNA-seq analysis was performed as previously described [44]. In brief, the total RNA was extracted using a Quick-RNA Microprep kit (ZYMO Research, Seattle, USA) from both treated and untreated yeast cells. mRNA was purified from the input RNA material (3 μg) using poly T oligo-attached magnetic beads. The sequencing library was sequenced on the NovaSeq 6000 platform (Illumina, San Diego, USA) by Shanghai Personal Biotechnology Co., Ltd. Filtered reads were mapped to the reference genome MYA-3404 of *C. tropicalis* (GenBank: GCF_000006335.3). The uniquely mapped read counts were normalized, and the differentially expressed genes (DEGs) were identified using the fragments per kilobase of transcript per million mapped reads method with a false discovery rate adjusted to *P* < 0.05 and fold change values ≥ 2.0. Yeast samples from three independent biological replicates were analyzed.

### Proteomic detection based on data independent acquisition (DIA) method

Strain ATCC750 and SLT34 were incubated at 37 °C for 1 h, with or without caspofungin treatment. After protein extraction from each sample, the BCA method was used for protein quantification, followed by trypsin digestion. The resulting peptides from the digested samples were desalted using a C18 Cartridge. An appropriate amount of iRT standard peptides was added to the tryptic peptides of each sample. DIA mass spectrometry analysis was conducted using a Orbitrap Astral high-resolution mass spectrometer (Thermo Fisher Scientific, USA). DIA data was processed with the DIA-NN software. In the screening for differentially expressed proteins between comparison groups, proteins with a fold change (FC) > 1.5 and a *P* value < 0.05 were selected. GO and KEGG annotations were applied to the target proteins using Blast2GO (version: BLASTP 2.8.0) and KOBAS (version: KOBAS 3.0) software, respectively. The workflow was supported by Shanghai Personal Biotechnology Co., Ltd. Yeast samples from three independent biological replicates were analyzed in this study.

### Gene expression analysis using quantitative real-time PCR

Total RNA was extracted from yeast cells in the logarithmic growth phase using the Quick-RNA Microprep kit (ZYMO Research, USA). Complementary DNA (cDNA) was synthesized using the PrimeScript RT reagent kit (TaKaRa,Tokyo, Japan). Then the quantitative PCR was performed using the SYBR Premix Ex Taq II (Tli RNaseH Plus) kit with the primers listed in S6 Table. The relative mRNA expression levels of the target genes were calculated using the 2^-ΔΔCt^ method, with *ACT1* serving as the reference gene. Data for each gene were derived from three independent biological replicates.

### Plasmid and strain construction

The construction of the gene overexpression strain was carried out through homologous recombination. In brief, we inserted the *SAT1* cassette and the *TDH3* promoter between the 1000 bp sequence upstream and 1000 bp sequence downstream of the target gene's start codon. The entire construct, complete with the necessary restriction enzyme sites at both ends, was synthesized by Tsingke Biotechnology Co., Ltd. using standard gene synthesis techniques. Following restriction enzyme digestion, the gene synthesis product was cloned into the vector pUC57. Subsequently, the *ACE2* and *SCW11* genes, after being digested with NdeI/NheI, were introduced into the SLT9 and SLT34 strains. Similarly, the *ENG1* gene, digested with NheI/KpnI, was transformed into the same strains. Transformants resistant to nourseothricin were identified through colony PCR to verify that the *TDH3* promoter had accurately replaced the native promoters of the target genes. Finally, RT-qPCR was used to assess the expression changes of the target gene mRNA in the mutant strains, and strains with the highest increase in gene expression were selected for further experiments. The primers, plasmids and strains used here are listed in S6 Table.

### Statistical analysis

All statistical analyses were conducted using GraphPad Prism version 10.3, with specific details provided in the corresponding figure legends. For comparisons involving more than three groups with two independent variables, a two-way analysis of variance (ANOVA) was employed, followed by post-hoc multiple comparison tests. Survival curves of *Galleria mellonella* were compared utilizing the Log-rank (Mantel-Cox) test. Data are presented as the mean or mean ± standard deviation (SD) for continuous variables. A *P*-value of less than 0.05 (two-tailed) was considered to indicate statistical significance.

## Supporting information

S1 Fig Spot growth assays. Tolerant isolates and the standard strain ATCC750 were cultured overnight, washed in PBS, and were spotted with 10 fold serial dilutions onto YPD or YPD supplemented with 1ug/mL caspofungin or 1ug/mL micafungin. The plates were incubated at 37 °C for 2 days, and then pictures were taken.
(TIF)

S2 Fig Kaplan-Meier survival analysis of *G. mellonella* larvae (n = 20 per group) following infection with the tolerant strains and non-tolerant strains (SLT2 and SLT14). Caspofungin-treated groups received 0.5 μg/larva at 1-hour post-infection. Statistical analysis was performed using a log rank (Mantel-Cox) test. *P* values are displayed directly on the figures, with *P* < 0.05 considered statistically significant.
(TIF)

S3 Fig Comparative morphology of tolerant versus non-tolerant (SLT2 and SLT14) strains on YPD agar containing 1 μg/mL caspofungin. Cultures in the logarithmic growth phase were harvested from all strains by centrifugation, washed with PBS, and subsequently diluted for spreading onto YPD agar plates, both with and without the addition of 1 µg/mL caspofungin. Following a 48-hour incubation at 37 °C, photographs were taken.
(TIF)

S4 Fig Morphological comparison of caspofungin-treated tolerant versus non-tolerant (SLT2 and SLT14) strains. The scale bar represents 20 µm.
(TIF)

S5 Fig Multicellular aggregates were resistant to trypsin and proteinase K digestion but could be physically dispersed by ultrasonication. Aggregated cells were resuspended in PBS and then subjected to treatment with either PBS, 100 μg/mL proteinase K (TianGen Biotech, China), or 0.25% trypsin (Biochannel Biotech, China) at 37 °C for overnight incubation. The scale bar represents 20 µm.
(TIF)

S6 Fig Assessment of propidium iodide staining of yeasts from different strains under caspofungin exposure. Strains were cultivated to the logarithmic growth phase, followed by treatment with 1 μg/mL caspofungin for durations of 6 and 18 hours, respectively. Subsequent to rinsing, the cells were subjected to a 20-minute staining with propidium iodide (PI). The cells' staining status was microscopically assessed (top panel). Moreover, a quantitative analysis was conducted by counting 400 cells to ascertain the numbers of PI-stained and unstained cells (bottom panel). The scale bar represents 20 µm.
(TIF)

S7 Fig Morphological changes of *C. tropicalis* strains to caspofungin treatment in a macrophage co-culture system. The murine macrophage cell line RAW 264.7 was co-cultured with the strains at a multiplicity of infection (MOI) of 1. The co-culture system was supplemented with 1 μg/mL caspofungin. Following incubation periods of 24 hours and 48 hours at 37 °C, the samples were stained with calcofluor white (CFW) and subsequently examined using a fluorescence microscope. The scale bar represents 20 µm.
(TIF)

S8 Fig The chromatogram of the monosaccharide composition of the cell wall as determined by HPAEC-PAD. (TIF)

S9 Fig Genetic variation analysis in tolerant and non-tolerant strains utilizing whole genome re-sequencing data. Utilizing the R package circlize, we generate circular plots to depict whole-genome SNP (A, left panel) and indel (B, left panel) variation data. The innermost ring illustrates the gene density across the reference genome of *C. tropicalis* MYA3404. Encompassing that, the second through eighth circles sequentially represent the genomic variation profiles for strains ATCC750, SLT14, SLT13, SLT9, SLT21, SLT22, and SLT34. The depth of color in these rings is indicative of the quantity of genetic variations. The outermost ring provides an overview of the sequence ID and the length of the reference genome. Afterward, we performed an intersection analysis on the homozygous gene variation data of tolerant strains and subsequently carried out GO and KEGG analyses for the identified genes. We present the top-ranking terms from the enrichment analysis results of both SNP (A, right panel) and indel (B, right panel) variation genes.
(TIF)

S10 Fig The effect of different concentrations of nikkomycin Z on the survival of tolerant strains and the strain ATCC750 under caspofungin stress. The data are presented as mean ± SD and are representative of three biological replicates. Statistical analysis was performed using a two-way ANOVA with Tukey's post-hoc test. *, *P* < 0.05; **, *P* < 0.01; ****, *P* < 0.0001.
(TIF)

S11 Fig mRNA expression dynamics of cell division-related genes in tolerant strains and ATCC750 strain during caspofungin treatment. The strains were cultured to the logarithmic growth phase and then treated with 1 μg/mL caspofungin for 0.5, 2, and 4 hours, respectively. The mRNA expression changes of *ACE2* (A), *IQG1* (B), *ENG1* (C), and *SCW11* (D) genes were quantified using the RT-qPCR method. The results were normalized to the expression levels of the control gene *ACT1* and are presented as relative values compared to the untreated ATCC750 strain. Data are expressed as the mean ± standard deviation (SD). Statistical analysis was performed using a two-way ANOVA with Tukey's post-hoc test. *, *P* < 0.05; **, *P* < 0.01; ***, *P* < 0.001; ****, *P* < 0.0001; ns, no significance.
(TIF)

S12 Fig Spot growth assays. Strains were cultured to the logarithmic growth phase and adjusted to a density of 2 × 10^5^ CFU/mL. Subsequently, they were exposed to 1 μg/mL caspofungin along with a range of FK506 concentrations for a duration of 24 h. Following centrifugation and washing, the cells were resuspended in PBS and subjected to 10-fold serial dilution. Aliquots of 5 μL from each dilution were then spotted onto YPD agar plates and incubated for 24 hours before being photographed.
(TIF)

S13 Fig FK506 significantly reduces the tolerance of all remaining tolerant strains to caspofungin. (A) FK506 completely inhibits colony formation of tolerant strains on YPD agar plates containing 1μg/mL caspofungin. The strains were cultured to the logarithmic phase, harvested, and diluted to appropriate concentrations before being spread onto caspofungin-supplemented YPD plates. After incubation at 37 °C for 48 h, photographs were taken. (B) FK506 significantly suppresses the survival of tolerant strains in YPD liquid medium with 1 μg/mL caspofungin. Following growth to the logarithmic phase, strains were adjusted to a density of 2 × 10^5^ CFU/mL in caspofungin-supplemented YPD medium and incubated at 37 °C for 24 h. The yeast suspension was then diluted and 100 μL was spread onto YPD plates. After a 48-h incubation, CFUs were counted, and survival percentages were calculated relative to the untreated control group. Statistical significance was determined using two-way ANOVA with Tukey's test. ****, *P* < 0.0001.
(TIF)

S14 Fig Cyclosporin A significantly reduces the survival rate and completely inhibits the formation of multicellular aggregates in tolerant strains under caspofungin stress. (A) Cyclosporin A decreases the survival rate of tolerant strains under caspofungin. The strains were grown to the logarithmic phase, diluted to a concentration of 2 × 10^5^ CFU/mL, and treated with caspofungin and various concentrations of cyclosporin A for 24 h. The cells were then washed, resuspended in PBS, diluted to appropriate concentrations, spread on YPD plates, and incubated at 37 °C. After a 48-h incubation, CFUs were counted, and survival percentages were calculated relative to the untreated control group. Statistical significance was determined using two-way ANOVA with Tukey's test. ****, *P* < 0.0001. (B) Cyclosporin A completely inhibits the formation of multicellular aggregates of tolerant strains under caspofungin stress. The strains were grown to the logarithmic phase, diluted to an optical density (OD) of 2, and treated with caspofungin and cyclosporin A for 24 h. The cells were then fixed with 4% paraformaldehyde, washed, resuspended in PBS, and stained with calcofluor white (CFW). Cellular morphologies were observed under a fluorescence microscope. The scale bar represents 20 µm.
(TIF)

S1 Table Detailed clinical information and strain chracteritics on *Candida tropicalis* isolates of bloodstream infection. (XLSX)

S2 Table Differentially gene expression profiling and statistical analysis of RNA-seq data. (XLSX)

S3 Table Integrative analysis of transcriptome and whole genome sequencing data. (XLSX)

S4 Table Differentially expressed proteins and statistical analysis identified by data-independent acquisition (DIA) proteomics. (XLSX)

S5 Table Integrative analysis of differentially expressed genes from transcriptomic data and differentially expressed proteins from proteomic data. (XLSX)

S6 Table Primers, plasmids and strains used in this study. (XLSX)
