# Correction: Adaptive morphological changes link to poor clinical outcomes by conferring echinocandin tolerance in *Candida tropicalis*

**DOI:** 10.1371/journal.ppat.1014188

**Published:** 2026-05-05

**Authors:** Yongqin Wu, Yun Zou, Yuanyuan Dai, Huaiwei Lu, Wei Zhang, Wenjiao Chang, Ying Wang, Zhengchao Nie, Yuanyuan Wang, Xiaohua Jiang

Following publication of the article [1] the authors noted that the Fig 2B 6 h ATCC750 panel and the Fig S3 STL6 plate panel are incorrect. An updated Fig 2 and an updated Fig S3 (S1 File) presenting the correct panels are provided with this notice.

The individual-level underlying data for Figs 1, 3–6, S2, S6, S10, S11, S13 and S14 were not originally provided with [1]. The authors have provided these data as S2 File.

The authors apologize for the errors in the published article.

**Fig 2:**
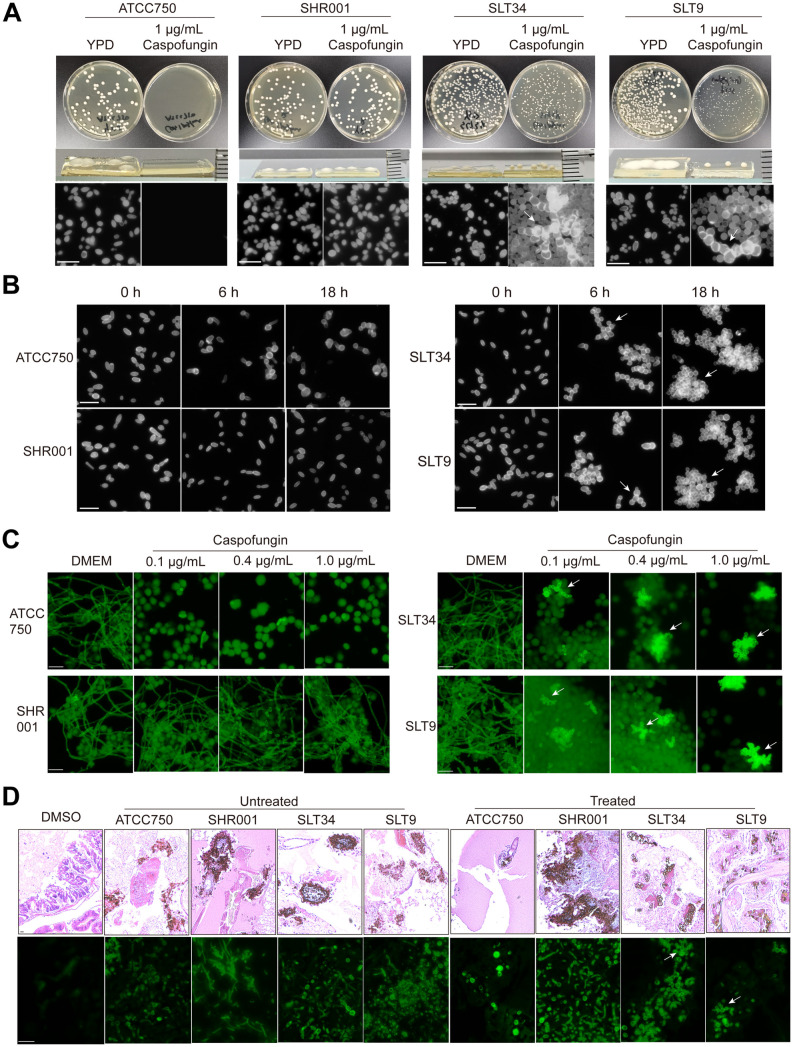
Multicellular aggregation in tolerant strains following caspofungin exposure, as observed *in vitro* and *in vivo.* **(A)** Cultures at the logarithmic growth phase of all strains were pelleted by centrifugation, rinsed with PBS, and then diluted before being spread onto YPD agar plates with and without 1ug/mL caspofungin. After a 48-hour incubation at 37°C, images were captured. Subsequently, individual colonies were suspended in PBS, and stained with Calcofluor White (CFW) for microscopic analysis. ATCC750 is the reference strain, SHR001 is characterized as caspofungin-resistant, and SLT34 and SLT9 are classified as tolerant strains. The scale bar represents 20 µm. **(B)** Each strain was cultured in YPD liquid medium containing caspofungin, with an initial optical density (OD) of 2, and incubated at 37 °C with shaking. At 0, 6, and 18 hours’ post-drug exposure, aliquots of the culture were taken, fixed with 4% paraformaldehyde, then stained with CFW, and observed under a fluorescence microscope. **(C)** The cell line RAW 264.7 was co-cultured with the strains at a multiplicity of infection (MOI) of 10, and various concentrations of caspofungin were added to the co-culture system. After an 18-hour incubation period at 37 °C, the samples were stained with CFW and then examined under a fluorescence microscope. **(D)** After a 48-hour infection of *G. mellonella* larvae with the strains, histopathological examination was performed. Tissue sections were stained using haematoxylineosin (HE) to assess general morphology and CFW to highlight specific cellular structures. White arrows indicate multicellular aggregate morphology. The scale bar represents 20 µm.

## Supporting information

S1 File Updated S3 Fig. Comparative morphology of tolerant versus non-tolerant (SLT2 and SLT14) strains on YPD agar containing 1 μg/mL caspofungin. Cultures in the logarithmic growth phase were harvested from all strains by centrifugation, washed with PBS, and subsequently diluted for spreading onto YPD agar plates, both with and without the addition of 1 µg/mL caspofungin. Following a 48-hour incubation at 37 °C, photographs were taken.
(TIF)

S2 File Source Data. (XLSX)
