# Supplementary figures and images for: Adaptive morphological changes link to poor clinical outcomes by conferring echinocandin tolerance in Candida tropicalis
Source: PLoS Pathog. 2025 May 27;21(5):e1013220. doi: 10.1371/journal.ppat.1013220 (PMC12140413; doi:10.1371/journal.ppat.1013220)

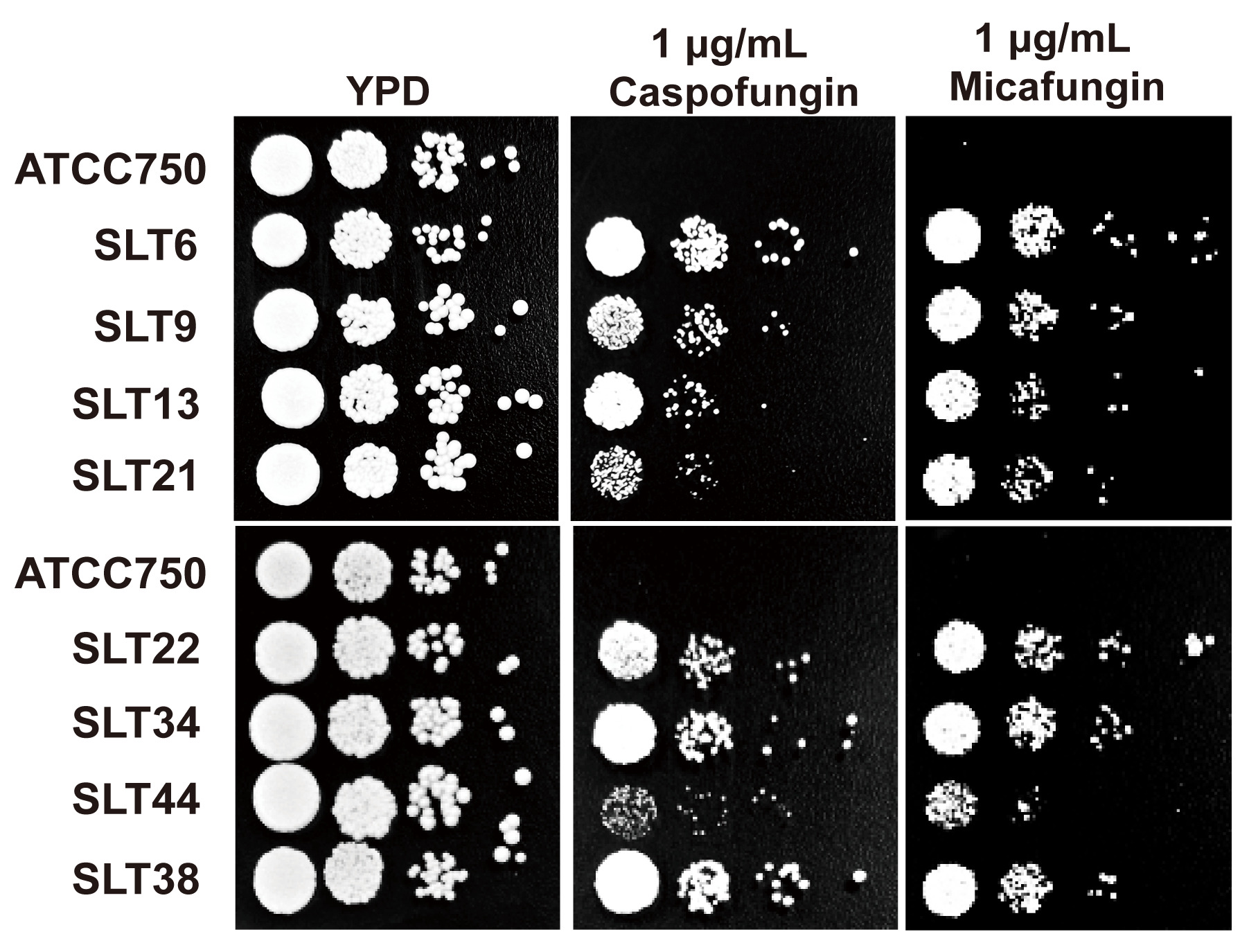

Supplement: S1 Fig — Tolerant isolates and the standard strain ATCC750 were cultured overnight, washed in PBS, and were spotted with 10 fold serial dilutions onto YPD or YPD supplemented with 1ug/mL caspofungin or 1ug/mL micafungin. The plates were incubated at 37 °C for 2 days, and then pictures were taken. (TIF) [file ppat.1013220.s001.tif]

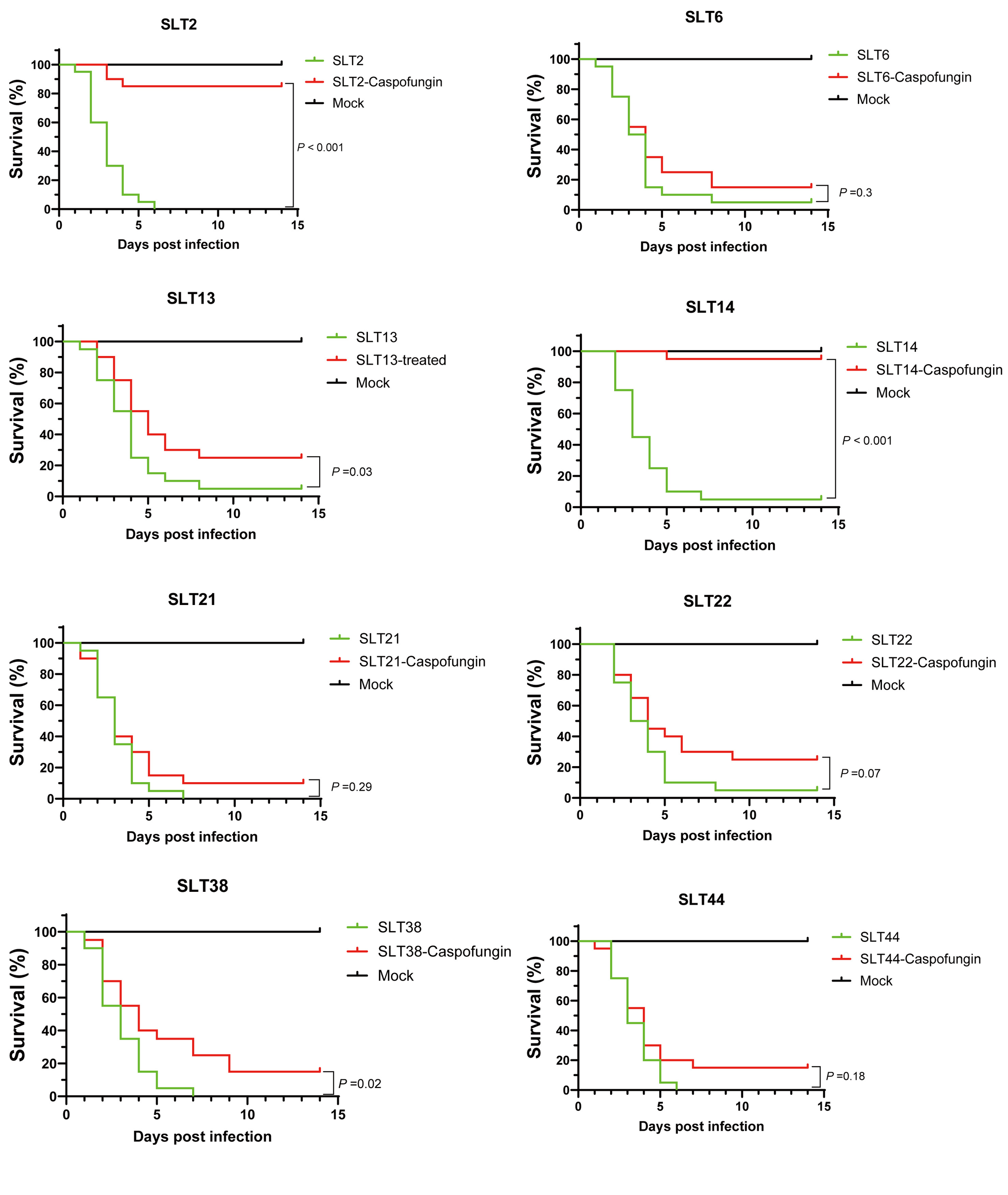

Supplement: S2 Fig — Caspofungin-treated groups received 0.5 μg/larva at 1-hour post-infection. Statistical analysis was performed using a log rank (Mantel-Cox) test. P values are displayed directly on the figures, with P < 0.05 considered statistically significant. (TIF) [file ppat.1013220.s002.tif]

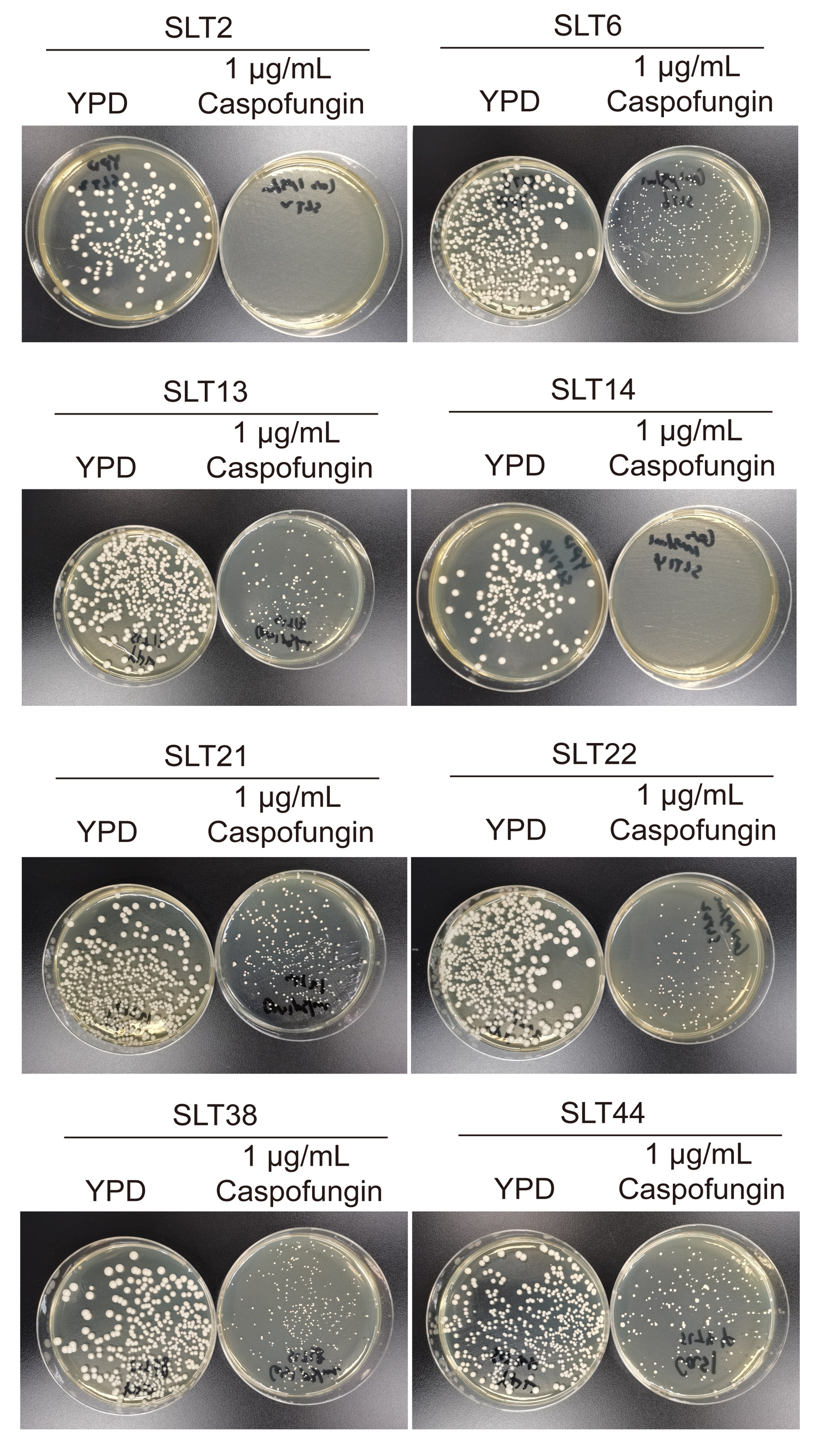

Supplement: S3 Fig — Cultures in the logarithmic growth phase were harvested from all strains by centrifugation, washed with PBS, and subsequently diluted for spreading onto YPD agar plates, both with and without the addition of 1 µg/mL caspofungin. Following a 48-hour incubation at 37 °C, photographs were taken. (TIF) [file ppat.1013220.s003.tif]

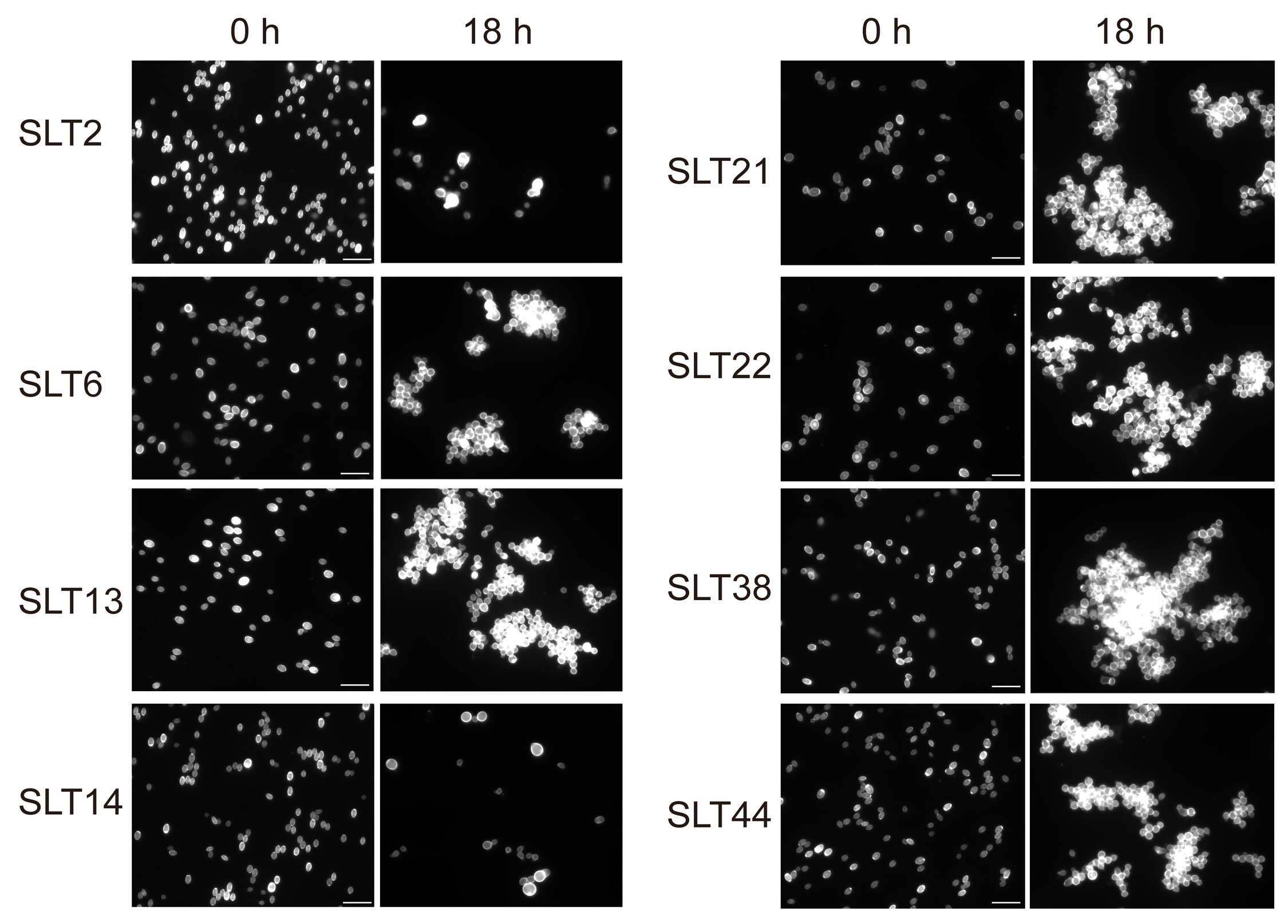

Supplement: S4 Fig — The scale bar represents 20 µm. (TIF) [file ppat.1013220.s004.tif]

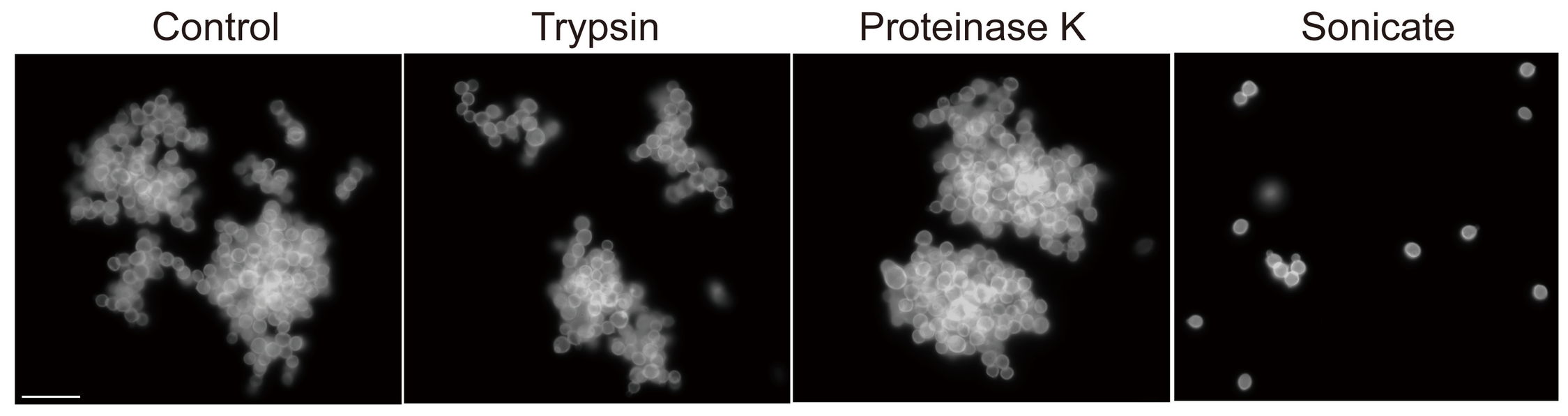

Supplement: S5 Fig — Aggregated cells were resuspended in PBS and then subjected to treatment with either PBS, 100 μg/mL proteinase K (TianGen Biotech, China), or 0.25% trypsin (Biochannel Biotech, China) at 37 °C for overnight incubation. The scale bar represents 20 µm. (TIF) [file ppat.1013220.s005.tif]

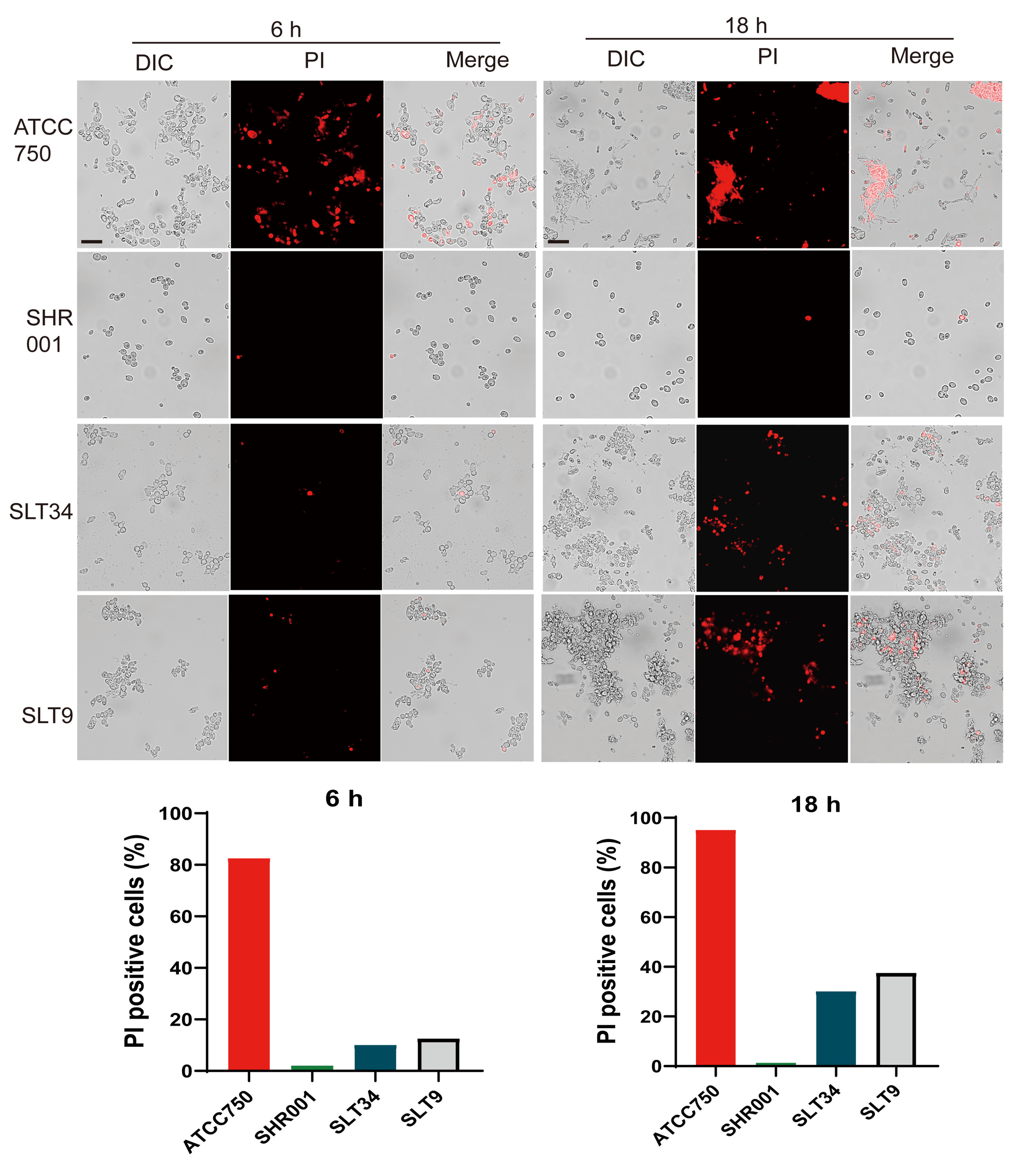

Supplement: S6 Fig — Strains were cultivated to the logarithmic growth phase, followed by treatment with 1 μg/mL caspofungin for durations of 6 and 18 hours, respectively. Subsequent to rinsing, the cells were subjected to a 20-minute staining with propidium iodide (PI). The cells' staining status was microscopically assessed (top panel). Moreover, a quantitative analysis was conducted by counting 400 cells to ascertain the numbers of PI-stained and unstained cells (bottom panel). The scale bar represents 20 µm. (TIF) [file ppat.1013220.s006.tif]

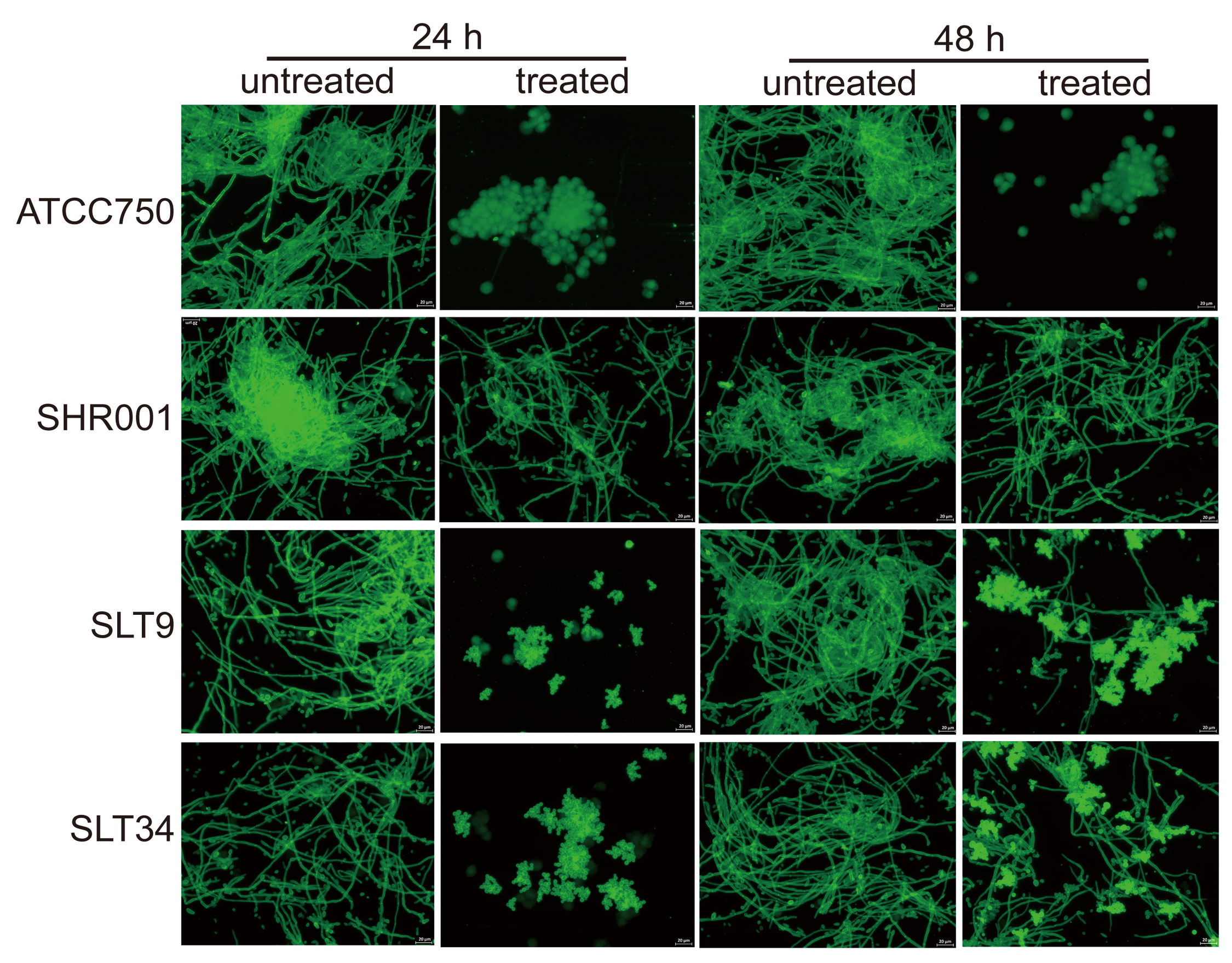

Supplement: S7 Fig — The murine macrophage cell line RAW 264.7 was co-cultured with the strains at a multiplicity of infection (MOI) of 1. The co-culture system was supplemented with 1 μg/mL caspofungin. Following incubation periods of 24 hours and 48 hours at 37 °C, the samples were stained with calcofluor white (CFW) and subsequently examined using a fluorescence microscope. The scale bar represents 20 µm. (TIF) [file ppat.1013220.s007.tif]

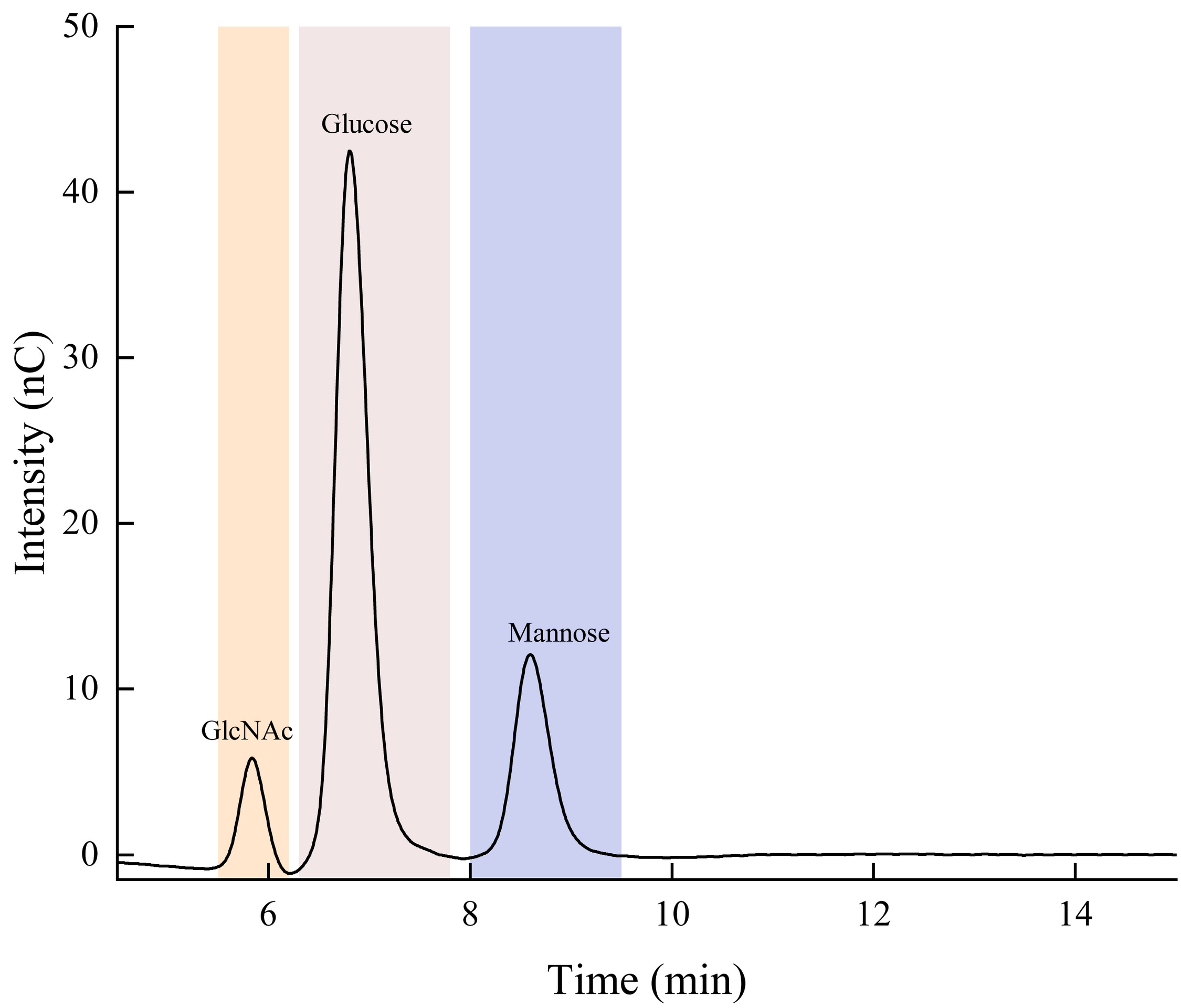

Supplement: S8 Fig — (TIF) [file ppat.1013220.s008.tif]

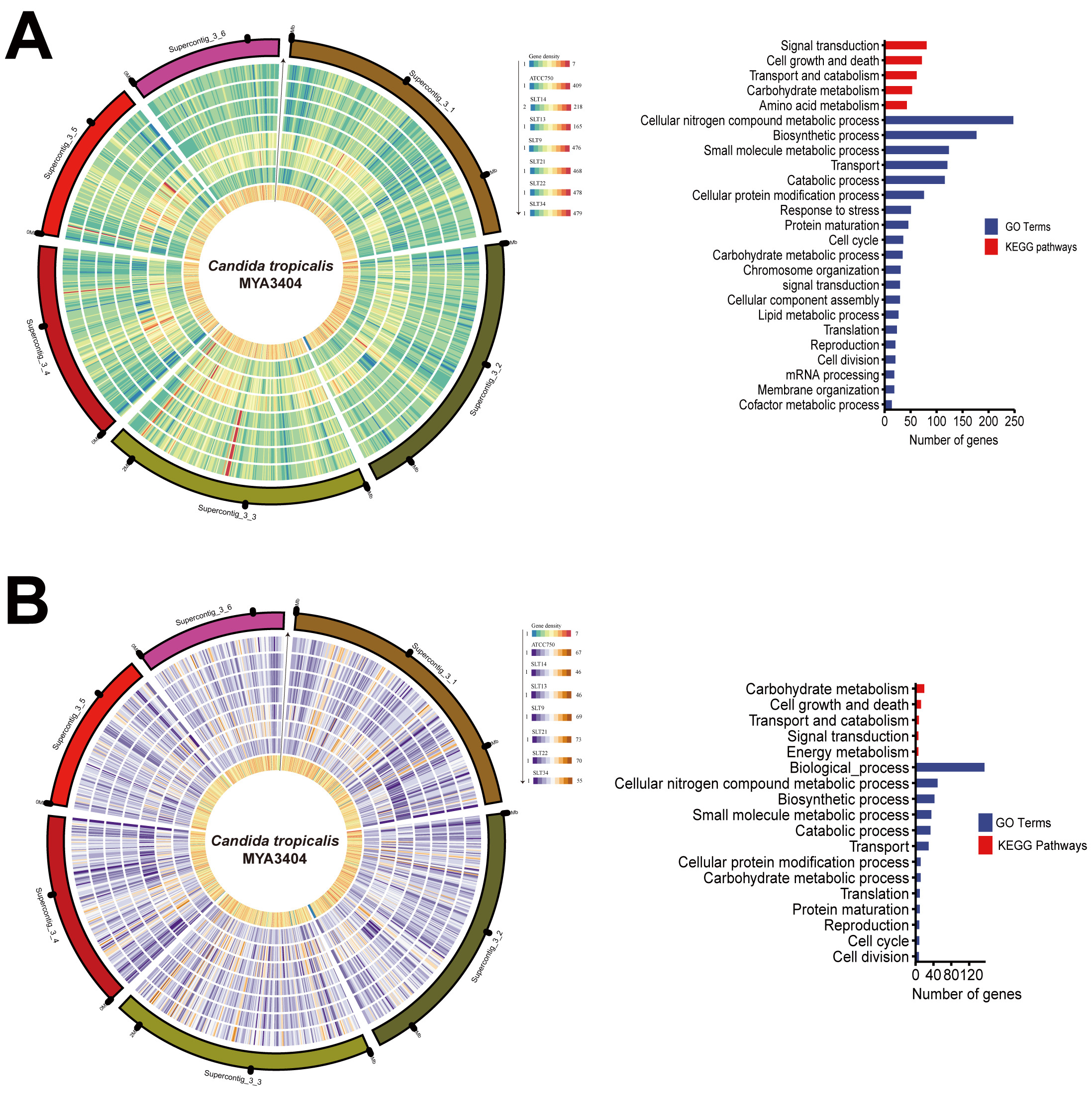

Supplement: S9 Fig — Utilizing the R package circlize, we generate circular plots to depict whole-genome SNP (A, left panel) and indel (B, left panel) variation data. The innermost ring illustrates the gene density across the reference genome of C. tropicalis MYA3404. Encompassing that, the second through eighth circles sequentially represent the genomic variation profiles for strains ATCC750, SLT14, SLT13, SLT9, SLT21, SLT22, and SLT34. The depth of color in these rings is indicative of the quantity of genetic variations. The outermost ring provides an overview of the sequence ID and the length of the reference genome. Afterward, we performed an intersection analysis on the homozygous gene variation data of tolerant strains and subsequently carried out GO and KEGG analyses for the identified genes. We present the top-ranking terms from the enrichment analysis results of both SNP (A, right panel) and indel (B, right panel) variation genes. (TIF) [file ppat.1013220.s009.tif]

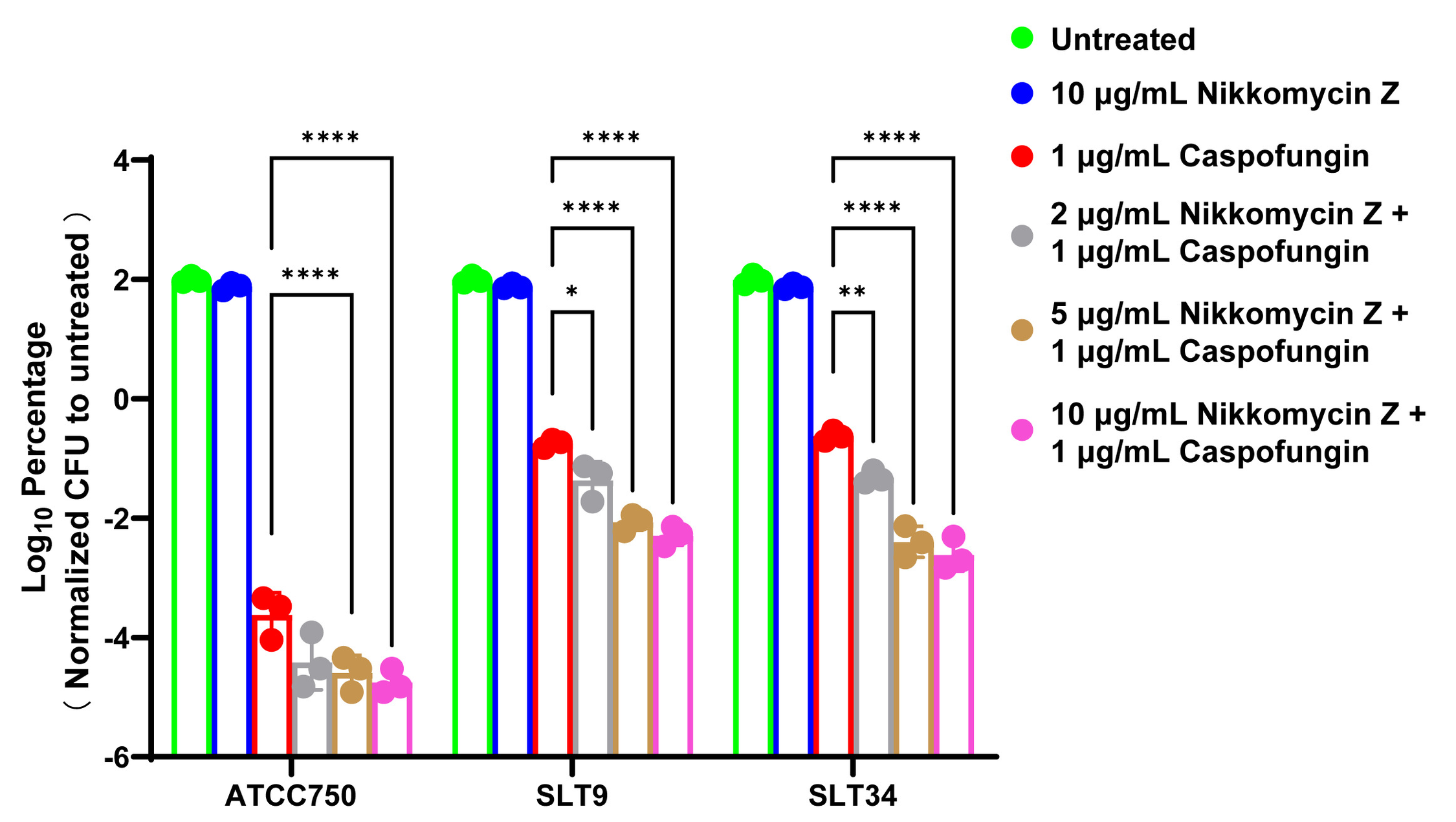

Supplement: S10 Fig — The data are presented as mean ± SD and are representative of three biological replicates. Statistical analysis was performed using a two-way ANOVA with Tukey's post-hoc test. *, P < 0.05; **, P < 0.01; ****, P < 0.0001. (TIF) [file ppat.1013220.s010.tif]

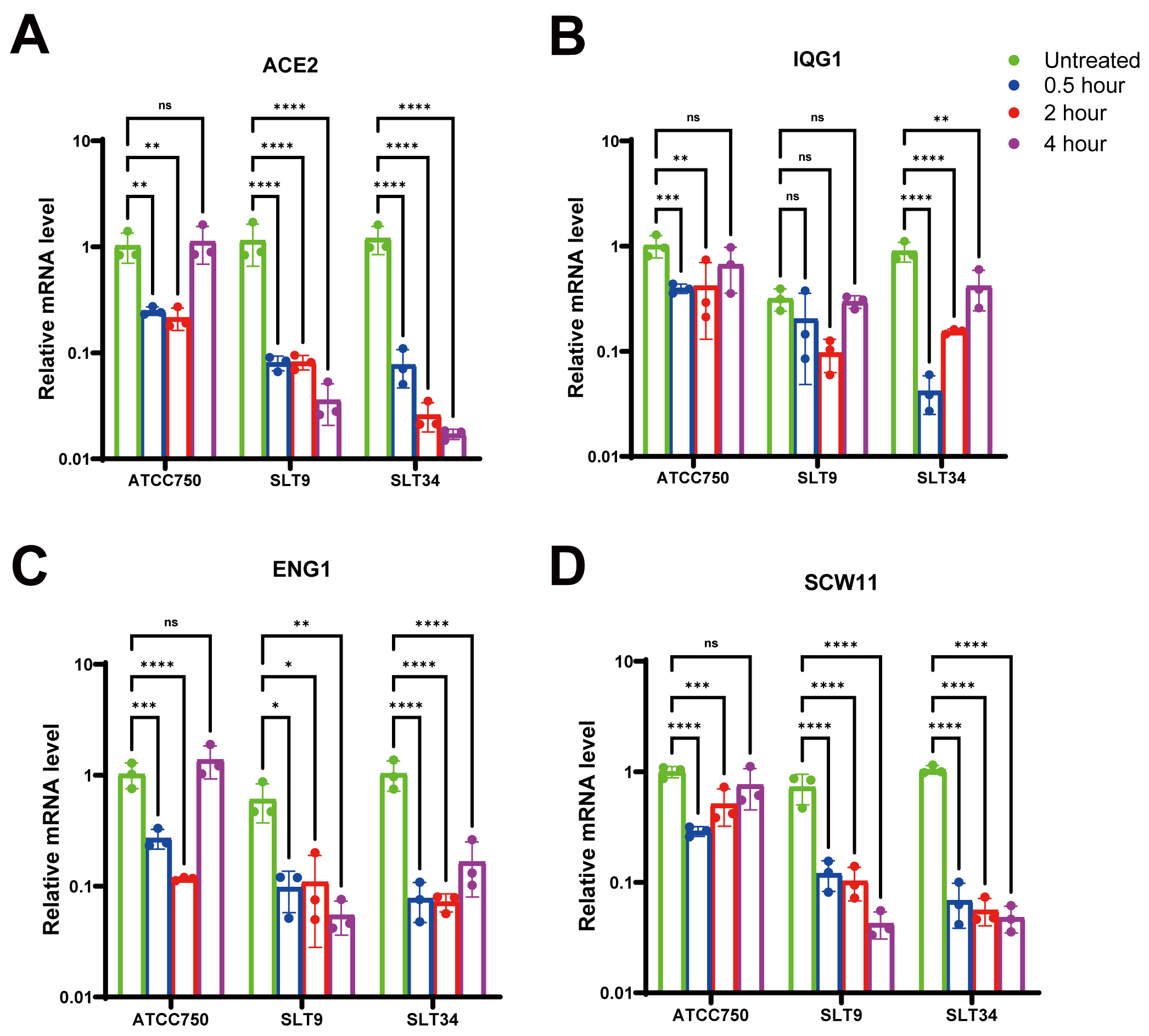

Supplement: S11 Fig — The strains were cultured to the logarithmic growth phase and then treated with 1 μg/mL caspofungin for 0.5, 2, and 4 hours, respectively. The mRNA expression changes of ACE2 (A), IQG1 (B), ENG1 (C), and SCW11 (D) genes were quantified using the RT-qPCR method. The results were normalized to the expression levels of the control gene ACT1 and are presented as relative values compared to the untreated ATCC750 strain. Data are expressed as the mean ± standard deviation (SD). Statistical analysis was performed using a two-way ANOVA with Tukey's post-hoc test. *, P < 0.05; **, P < 0.01; ***, P < 0.001; ****, P < 0.0001; ns, no significance. (TIF) [file ppat.1013220.s011.tif]

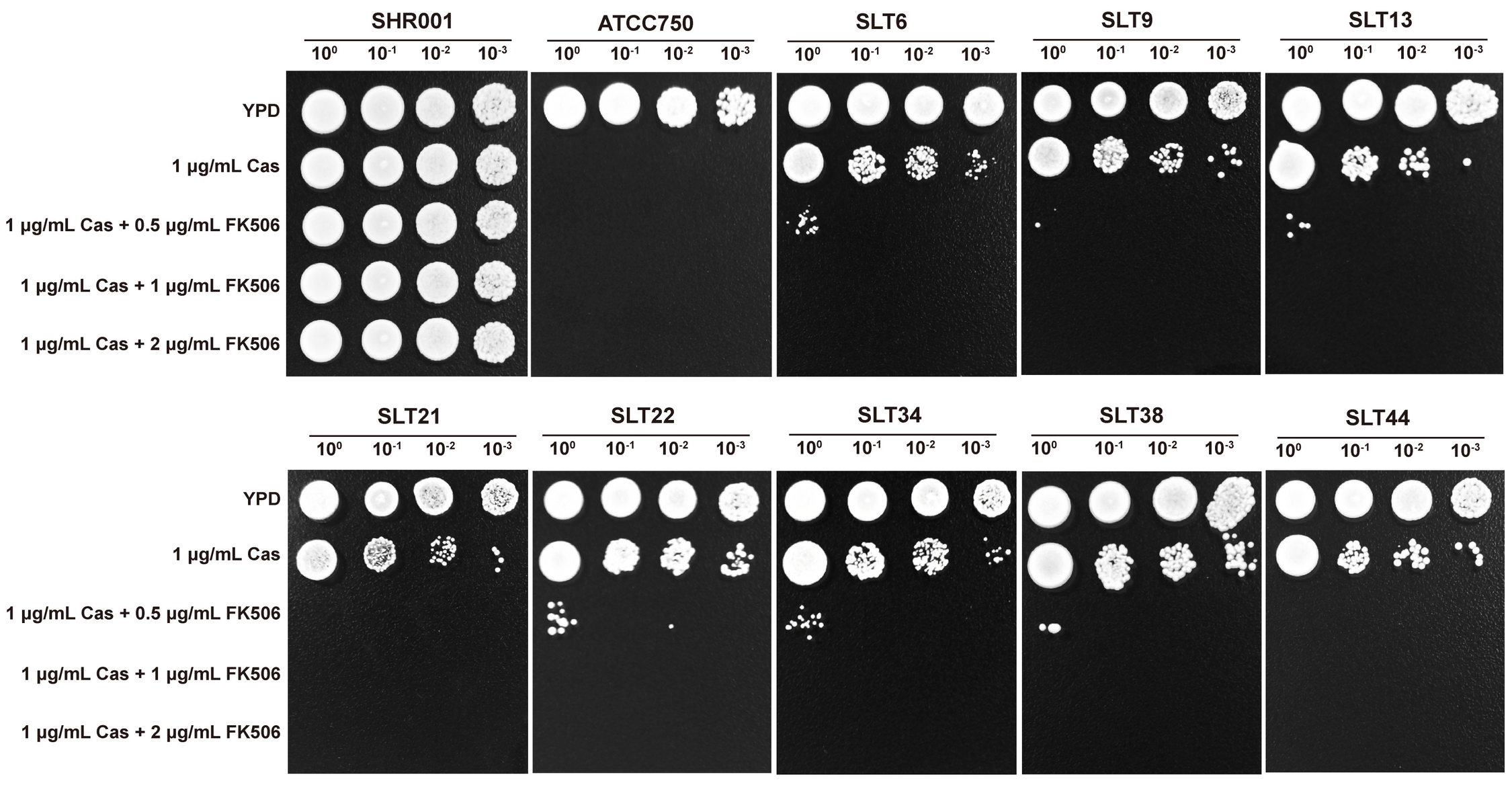

Supplement: S12 Fig — Strains were cultured to the logarithmic growth phase and adjusted to a density of 2 × 10^5^ CFU/mL. Subsequently, they were exposed to 1 μg/mL caspofungin along with a range of FK506 concentrations for a duration of 24 h. Following centrifugation and washing, the cells were resuspended in PBS and subjected to 10-fold serial dilution. Aliquots of 5 μL from each dilution were then spotted onto YPD agar plates and incubated for 24 hours before being photographed. (TIF) [file ppat.1013220.s012.tif]

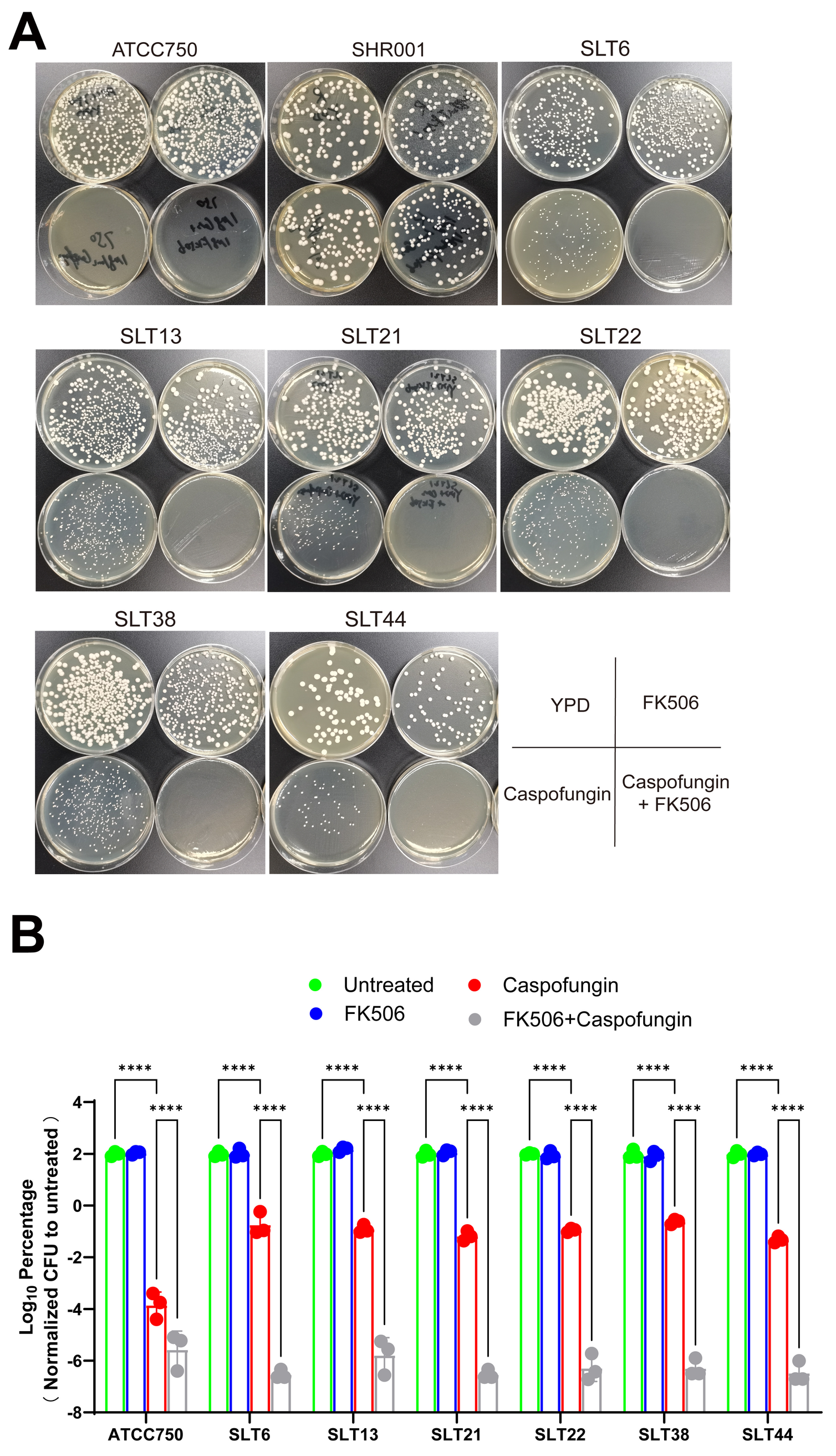

Supplement: S13 Fig — (A) FK506 completely inhibits colony formation of tolerant strains on YPD agar plates containing 1μg/mL caspofungin. The strains were cultured to the logarithmic phase, harvested, and diluted to appropriate concentrations before being spread onto caspofungin-supplemented YPD plates. After incubation at 37 °C for 48 h, photographs were taken. (B) FK506 significantly suppresses the survival of tolerant strains in YPD liquid medium with 1 μg/mL caspofungin. Following growth to the logarithmic phase, strains were adjusted to a density of 2 × 10^5^ CFU/mL in caspofungin-supplemented YPD medium and incubated at 37 °C for 24 h. The yeast suspension was then diluted and 100 μL was spread onto YPD plates. After a 48-h incubation, CFUs were counted, and survival percentages were calculated relative to the untreated control group. Statistical significance was determined using two-way ANOVA with Tukey's test. ****, P < 0.0001. (TIF) [file ppat.1013220.s013.tif]

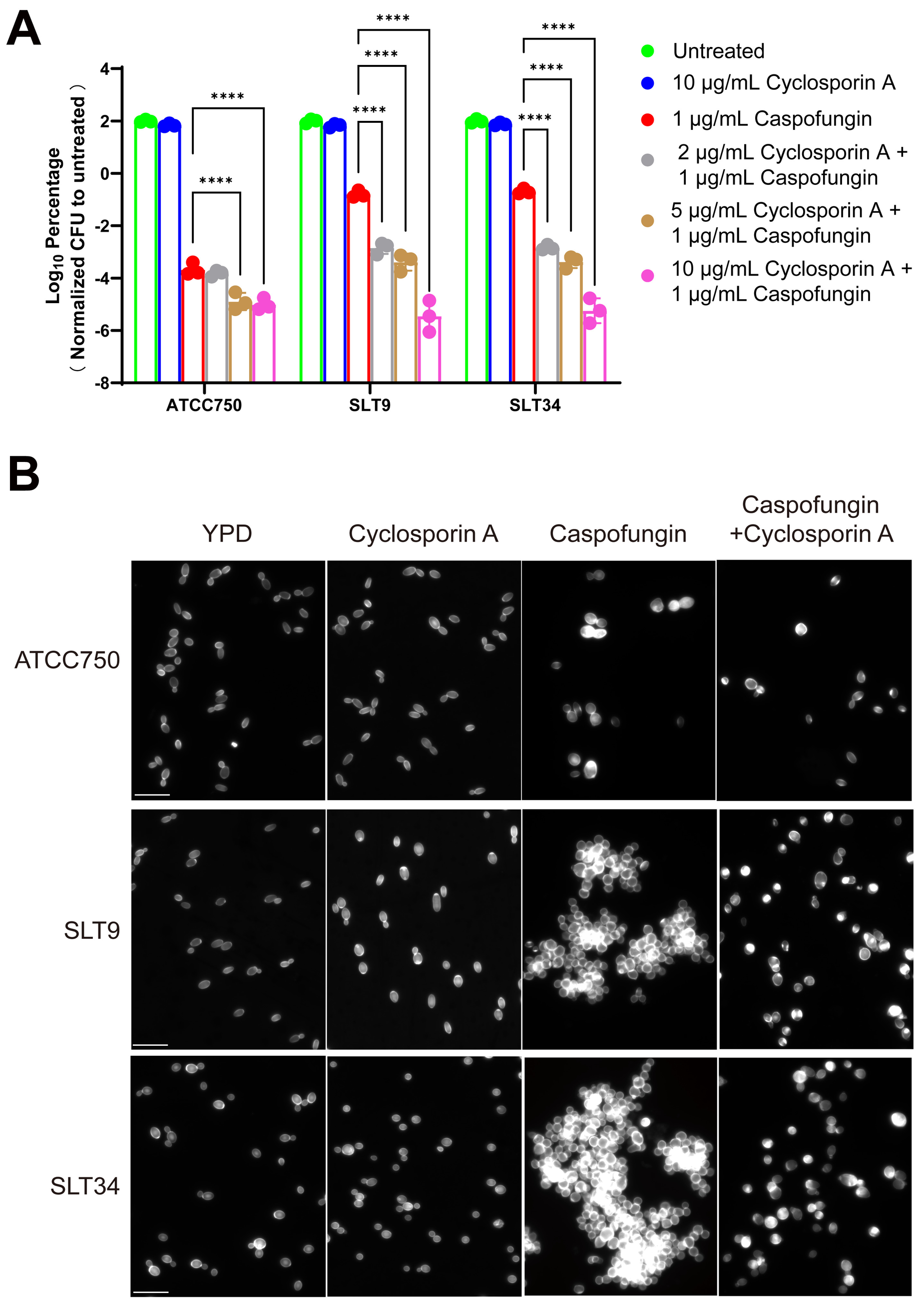

Supplement: S14 Fig — (A) Cyclosporin A decreases the survival rate of tolerant strains under caspofungin. The strains were grown to the logarithmic phase, diluted to a concentration of 2 × 10^5^ CFU/mL, and treated with caspofungin and various concentrations of cyclosporin A for 24 h. The cells were then washed, resuspended in PBS, diluted to appropriate concentrations, spread on YPD plates, and incubated at 37 °C. After a 48-h incubation, CFUs were counted, and survival percentages were calculated relative to the untreated control group. Statistical significance was determined using two-way ANOVA with Tukey's test. ****, P < 0.0001. (B) Cyclosporin A completely inhibits the formation of multicellular aggregates of tolerant strains under caspofungin stress. The strains were grown to the logarithmic phase, diluted to an optical density (OD) of 2, and treated with caspofungin and cyclosporin A for 24 h. The cells were then fixed with 4% paraformaldehyde, washed, resuspended in PBS, and stained with calcofluor white (CFW). Cellular morphologies were observed under a fluorescence microscope. The scale bar represents 20 µm. (TIF) [file ppat.1013220.s014.tif]
