# Supplementary figures and images for: Correction: Adaptive morphological changes link to poor clinical outcomes by conferring echinocandin tolerance in Candida tropicalis
Source: PLoS Pathog. 2026 May 5;22(5):e1014188. doi: 10.1371/journal.ppat.1014188 (PMC13143059; doi:10.1371/journal.ppat.1014188)

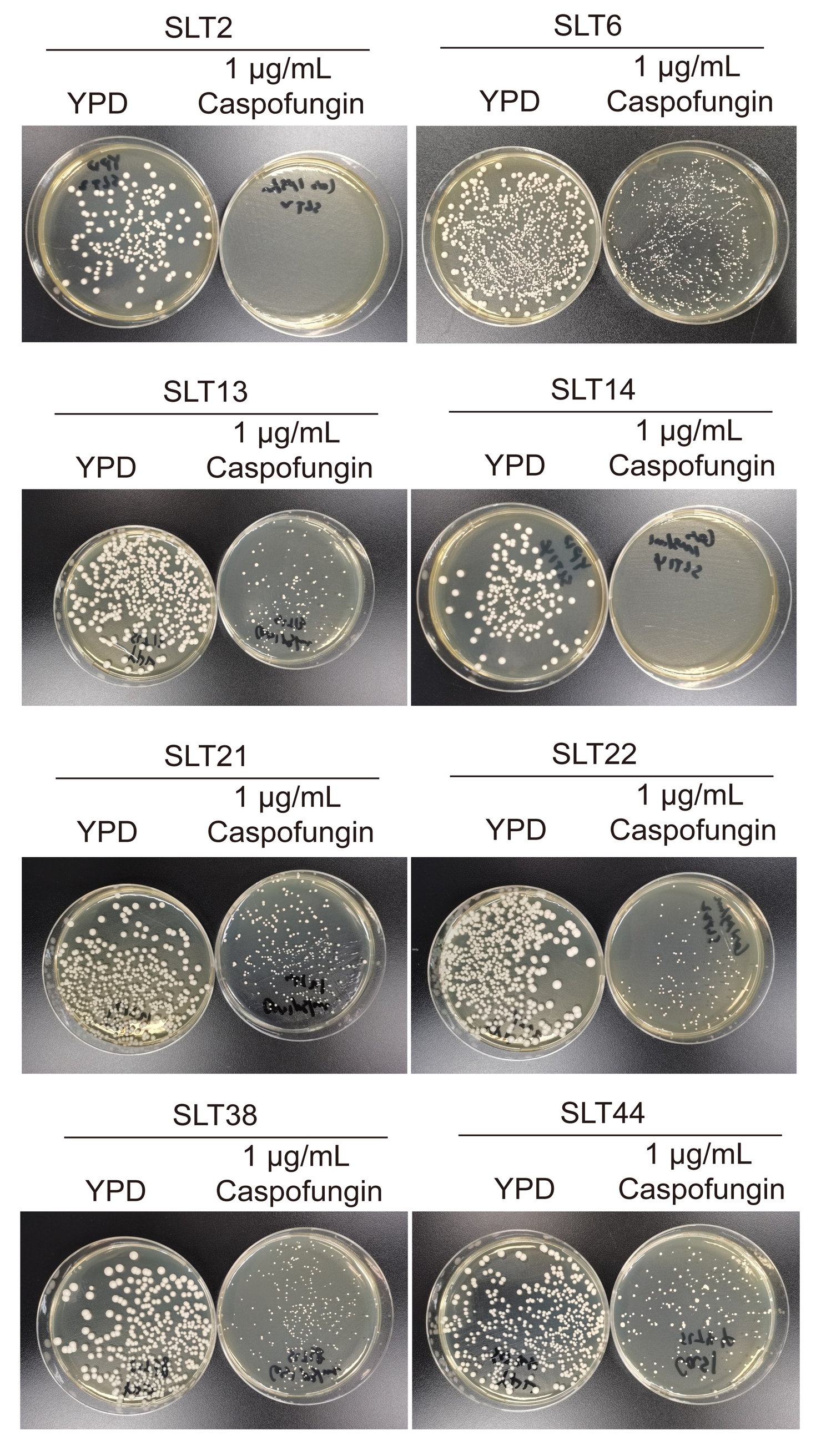

Supplement: S1 File — Comparative morphology of tolerant versus non-tolerant (SLT2 and SLT14) strains on YPD agar containing 1 μg/mL caspofungin. Cultures in the logarithmic growth phase were harvested from all strains by centrifugation, washed with PBS, and subsequently diluted for spreading onto YPD agar plates, both with and without the addition of 1 µg/mL caspofungin. Following a 48-hour incubation at 37 °C, photographs were taken. (TIF) [file ppat.1014188.s001.tif]
